# Features and Colonization Strategies of *Enterococcus faecalis* in the Gut of *Bombyx mori*

**DOI:** 10.3389/fmicb.2022.921330

**Published:** 2022-06-24

**Authors:** Xiancui Zhang, Huihui Feng, Jintao He, Abrar Muhammad, Fan Zhang, Xingmeng Lu

**Affiliations:** ^1^College of Animal Sciences, Institute of Sericulture and Apiculture, Zhejiang University, Hangzhou, China; ^2^Key Laboratory of Animal Resistance Biology of Shandong Province, College of Life Science, Shandong Normal University, Jinan, China

**Keywords:** *Bombyx mori*, insect, gut microbiota, *Enterococcus*, colonization strategies, whole-genome sequencing, bacteriocin, gene expression

## Abstract

The complex gut microbiome is a malleable microbial community that can undergo remodeling in response to many factors, including the gut environment and microbial properties. *Enterococcus* has emerged as one of the predominant gut commensal bacterial and plays a fundamental role in the host physiology and health of the major economic agricultural insect, *Bombyx mori.* Although extensive research on gut structure and microbiome diversity has been carried out, how these microbial consortia are established in multifarious niches within the gut has not been well characterized to date. Here, an *Enterococcus* species that was stably associated with its host, the model organism *B. mori*, was identified in the larval gut. GFP–tagged *E. faecalis* LX10 was constructed as a model bacterium to track the colonization mechanism in the intestine of *B. mori*. The results revealed that the minimum and optimum colonization results were obtained by feeding at doses of 10^5^ CFU/silkworm and 10^7^ CFU/silkworm, respectively, as confirmed by bioassays and fluorescence-activated cell sorting analyses (FACS). Furthermore, a comprehensive genome-wide exploration of signal sequences provided insight into the relevant colonization properties of *E. faecalis* LX10. *E. faecalis* LX10 grew well under alkaline conditions and stably reduced the intestinal pH through lactic acid production. Additionally, the genomic features responsible for lactic acid fermentation were characterized. We further expressed and purified *E. faecalis* bacteriocin and found that it was particularly effective against other gut bacteria, including *Enterococcus casselifavus*, *Enterococcus mundtii*, *Serratia marcescens*, *Bacillus amyloliquefaciens, and Escherichia coli.* In addition, the successful colonization of *E. faecalis* LX10 led to drastically increased expression of all adhesion genes (*znuA*, *lepB*, *hssA*, *adhE*, *EbpA*, and *Lap*), defense genes (*cspp, tagF*, and *esp*), regulation gene (*BfmRS*), secretion gene (*prkC*) and immune evasion genes (*patA* and *patB*), while the expression of iron acquisition genes (*ddpD* and *metN*) was largely unchanged or decreased. This work establishes an unprecedented conceptual model for understanding *B. mori*–gut microbiota interactions in an ecological context. Moreover, these results shed light on the molecular mechanisms of gut microbiota proliferation and colonization in the intestinal tract of this insect.

## Introduction

The insect gut is colonized by a complex bacterial community that governs a wide range of functions contributing to the host’s physiology, pathogen defense, development, and immune protection (Engel and Moran, 2013; Xiao et al., 2017). Thus, the gut microbiota may be recognized as a virtual “bacterial organ” that is integrated into the host’s biological system and fundamental to its health (Aksoy, 2018; Schretter, 2020). For instance, a comparison between aposymbiotic and symbiotic insects showed that the *Burkholderia* symbiont dramatically increases the fecundity, growth rate, and body size of stinkbugs by providing essential metabolites, and the insect host can also develop resistance against the insecticide fenitrothion (Kikuchi et al., 2007; Kikuchi and Fukatsu, 2014). The phytophagous leafminer *Phyllonorycter blancardella* (Lepidoptera) co-opts the bacterial endosymbiont *Wolbachia* to improve its nutritional and physiological environment (Kaiser et al., 2010). Furthermore, associations with specific microorganisms may improve environmental fitness or provide new opportunities for exploring new niches (Robinson et al., 2018). Although myriad microorganisms inhabit the guts of insects, many gut community members are transient, and it is sometimes difficult to create a resident microbiota due to various intrinsic factors (developmental stage, sex, genetics, gut pH, digestive enzymes, etc.) (Engel and Moran, 2013; Hammer et al., 2017).

The silkworm, *Bombyx mori*, is a powerful model organism for studying the bacterial colonization of the invertebrate intestine and for deciphering the interactions between the host and its gut microbiota (Chen et al., 2018, 2020). The midguts of silkworm larvae exhibit extremely high alkalinity, with pH levels of 10–11, and their digestive enzymes are adapted to the alkaline environment (Liang et al., 2018). Recent extensive surveys have revealed that these adverse conditions may support the growth of alkaline-resistant and facultative anaerobic microorganisms, such as Firmicutes, Planctomycetes, and Clostridium (Bignell et al., 2011; Yeruva et al., 2020). For example, *Enterococcus* (Firmicutes), which plays a crucial role in metabolic adaptability against pathogenic or plant toxins and anti-herbivore defense, was found to be one of the predominant gut microorganism of lepidopteran insects, including *B. mori*, *Helicoverpa zea*, and *Porthetria dispar* (Paniagua Voirol et al., 2018; Zhang et al., 2022). Some other insects harbor similar bacterial lineages in their alkaline guts (Egert et al., 2003; Broderick et al., 2004). Several physiological and biochemical characteristics of the gut environment influence the colonization of such symbionts, including the constant expulsion of intestinal contents *via* peristalsis, pH levels, reactive oxygen species, nutrient availability, digestive enzymes, and possibly antimicrobial agents (Siegel and Weiser, 2015). Thus, it appears likely that bacteria stably inhabiting the gut would have developed sophisticated mechanisms to resist these challenges.

The green fluorescent protein (GFP) of the jellyfish *Aequorea victoria*, first described in the 1970s, is a bright, stable molecular marker used in live-cell imaging to facilitate bacterial localization, isolation, and tracking in biological studies of several host species (Rizzo et al., 2009; Viswanathan et al., 2015). Genetically modified bacteria labeled with GFP or other fluorescent proteins have provided a unique and powerful tool for understanding the spatiotemporal dynamics and ecological interactions of the gut microbiota (Wang S. et al., 2017). Recently, similar research has been performed in *Pseudomonas*-zebrafish, *Photorhabdus*-nematode, *Burkholderia*-bean bug, and other model symbiotic systems (Rawls et al., 2007; Ciche et al., 2008; Kikuchi and Fukatsu, 2014). For example, the *Pseudomonas aeruginosa*: GFP transcriptional reporter strain is detectable in the *Caenorhabditis elegans* head and intestine regions using fluorescence microscopy (Sellegounder et al., 2019). Despite the recognized importance of gut-associated bacteria to *B. mori*, how and where its gut microbiota achieves colonization and the genetic and molecular bases of colonization are still open questions. In the current study, we constructed a genetically engineered strain of *E. faecalis* LX10 expressing GFP to visualize how the test organism adapted to the gut environment of silkworms. *E. faecalis* LX10 grew well under alkaline conditions and showed efficient lactic acid production. We further analyzed bacteriocin production and bacterial competition based on whole-genome sequencing. In addition, we quantified colonization-related genes following the growth of the bacterium in the gut using quantitative real-time PCR (RT–qPCR). The present study enhances our understanding of gut colonization by an indigenous bacterium and may promote the application of probiotic targeting regulation in agricultural and economic insects or for pest control of lepidopterans.

## Materials and Methods

### Sample Collection and Rearing

The *B. mori* Haoyue × Jingsong strain was hatched from eggs provided by the Silkworm Germplasm Bank at the College of Animal Sciences, Zhejiang University, China. Larvae were raised on fresh mulberry leaves at 25 ± 1°C under 70 ± 5% relative humidity and an alternating 14:10 h light/dark photoperiod.

### Survey and Characteristics of *Enterococcus* From the Gut of *Bombyx mori*

*Enterococcus* bacteria were isolated from the gut of normal *B. mori* larvae using *Enterococcus*-selective agar with 1% 2,3,5-triphenyl tetrazolium chloride (TTC) in the appropriate medium (Hopebio, Qingdao, China) (Supplementary Table 1). Random samples of bacterial colonies were picked from agar plates and subcultured at least three times before identification. Molecular analysis was performed by isolating genomic DNA and amplifying bacterial 16S rRNA genes using the 27 F/1492 R universal primers. The synthesis and sequencing of PCR products were performed by Sangon Biotech (Shanghai, China). The phylogenetic tree was constructed *via* the multiple sequence alignment of 16S rRNA genes performed with ClustalW and the neighbor-joining method in MEGA7 (7.0.14) software.

After anesthetizing the *B. mori* larvae on ice, the gut tissue was dissected from larvae of each developmental stage (1st–5th-instar larvae), transferred to a 2 mL sterile centrifuge tube containing 500 μL sterile PBS and then thoroughly homogenized. The number of gut *Enterococcus* bacteria in each sample was quantified by standard colony-forming unit (CFU) enumeration and absolute quantification *via* quantitative real-time PCR (qPCR). To determine CFU counts, each sample was serially diluted from 1 × 10^–1^ to 1 × 10^–6^ and plated, and the plates were incubated for 48 h at 30°C until colonies formed. Colonies were then manually counted (*n* = 10). For absolute quantification, total DNA was extracted from 1st–5th-instar larvae using a DNA kit (Epicenter, Madison, United States) following the manufacturer’s protocol. The final DNA concentration and purity were measured on a 2100 Bioanalyzer (Agilent, Palo Alto, United States), and DNA quality was then checked by 1% agarose gel electrophoresis. The absolute quantification of *Enterococcus* species, including *E. faecalis*, *E. mundtii*, and *E. casseliflavus*, was conducted by qPCR analysis (*n* = 10). The primers used in the qPCR assays are shown in Supplementary Table 2. The thermocycler conditions were as follows: 95°C for 5 min and 40 cycles of 95°C for 10 s, 60°C for 10 s, and 72°C for 20 s, followed by a final melting curve step (from 65 to 92°C, 0.5°C/s). A standard curve was generated from a mixed sample based on a series of 10–fold dilutions with the same reaction parameters, and the total 16S rRNA gene copies in each sample were calculated according to the standard curve.

For scanning electron microscopy (SEM) analysis, a *E. faecalis* LX10 culture was directly transferred to a fixative solution containing 2.5% glutaraldehyde (pH 7.4). Gut samples were then postfixed in 1% osmium tetroxide (OsO4) for 2 h, followed by dehydration in a graded ethanol series (70–100%) for 15 min. After washing with PBS, samples were dehydrated and critical point dried using carbon dioxide. Specimens were mounted on adhesive carbon films, coated with platinum (1 nm), and examined *via* SEM (Hitachi SU8000, Tokyo, Japan).

For fluorescence *in situ* hybridization (FISH) analysis, OCT-embedded gut tissue was frozen in a dry ice bath and sectioned at a 5 μm thickness on a freezing microtome (CryoStar, NX70, France). FISH was performed with a Cy3–labeled (GAAAGCGCCTTTCACTCTTATGC) *E. faecalis*–specific probe (red), and DNA was counterstained with DAPI (blue) in hybridization buffer (900 mM NaCl, 20% formamide, 20 mM Tris–HCl, 1% SDS) (Wellinghausen et al., 2007). Hybridization was achieved under previously described conditions (Koga et al., 2009).

### Construction of Green Fluorescent Protein–*Enterococcus faecalis* LX10

The bacterial strains and plasmids used in this study are listed in Supplementary Table 1. The GFP-containing plasmids were maintained in the *Escherichia coli* DH5α strain (TaKaRa, Dalian, China). *E. coli* strains were grown at 30°C in lysogenic broth (LB) medium. An enterococcal erythromycin ribosomal methylase (ermB) promoter drives green fluorescent protein 5 (GFP5) in the pTRKH3–ermGFP construct (Swinfield et al., 1990; Lizier et al., 2010) (Addgene plasmid 27169). pTRKH3–ermGFP was electroporated into *E. faecalis* LX10 competent cells using an Eppendorf Electroporator 2510 (Eppendorf, Hamburg, Germany) at 1.5 kV, 600 Ω, and 10 μF, with a pulse length of 3.6 ms. The electroporated cells were immediately mixed with 900 μL Todd Hewitt broth (THB), incubated for 1 h at 30°C, and plated on THB-agar containing erythromycin (5 μg/mL). Total DNA was extracted from GFP–tagged bacteria using a MasterPure™ complete DNA and RNA Purification Kit (Epicenter, Madison, United States) following the manufacturer’s protocol. The GFP gene (735 bp, GFP F/R) was amplified by PCR in a final volume of 25 μL using Taq DNA polymerase according to the manufacturer’s instructions (TaKaRa, Dalian, China). Images were captured with a confocal laser scanning microscope (CLSM) (LSM780, ZEISS, Germany). Growth curve analyses of *E. faecalis* LX10 and GFP–*E. faecalis* LX10 were conducted in 96-well honeycomb plates (Corning, United States), measuring the absorption at 600 nm for a total of 40 h in 4 h intervals.

### Analysis of Green Fluorescent Protein–*Enterococcus faecalis* Stability and Proliferation in the *Bombyx mori* Gut

Bacterial colonization challenges were performed with GFP–*E. faecalis* LX10. Each silkworm was fed on mulberry leaves (5 cm × 5 cm) that were previously treated with a GFP–*E. faecalis* LX10 suspension (1 × 10^3^, 1 × 10^5^, 1 × 10^7^, or 1 × 10^9^ CFU/mL) for 4 h. The same volume of sterilized PBS solution was used in the control groups (CK). The gut stability protocol conducted in a 90 mm petri dish was conducted with a single silkworm *per* dish, and feces were removed every 6 h. The gut tissue from each single gut was homogenized after 0, 1, 3, 5, or 7 days as described above.

Total gut GFP–*E. faecalis* counts were quantified by CFU enumeration, qPCR, and FACS. The CFUs were counted on each plate after a 48-h incubation period at 30°C (*n* = 10). Absolute quantification of the amount of GFP–*E. faecalis* 16S rRNA gene copies was conducted with the GFP F/R primer pair (Supplementary Table 2) (*n* = 10). For FACS, fluorescent *E. faecalis* bacteria were separated from the intestinal homogenate by filtration *via* 40 μm sterile cell strainers (Corning, United States) and diluted to the appropriate concentration. FACS analyses were performed using a BD FACSAria™ Fusion Cell Sorter (Becton Dickinson, Heidelberg, Germany). The filtrates were then separated into aliquots of 1 mL for each sample. GFP fluorescence was detected using a 488–nm blue laser line for signal excitation, and the wavelength was set to 493–578 nm. Intestinal bacteria were subjected to FACS to sort the GFP–positive and GFP–negative fractions. The corresponding control samples included a positive control (GFP–*E. faecalis*) and a negative control (intestinal homogenate without GFP) (*n* = 3). In addition, GFP fluorescence in the silkworm intestinal tract was visualized under an LSM780 confocal microscope (Zeiss) with oil immersion objectives after the silkworms had fed on GFP–*E. faecalis* for 5 days. Notably, the peritrophic membrane (PM) and intestinal gut digestive juice were mounted on slides and stored at 4°C until use. To determine if intestinal juice affects *E. faecalis* morphology, gut tissues were homogenized in a Precellys–24 homogenizer (Bertin Technologies, Aixen, France) at 5,500 × g for 20 s, centrifuged briefly at 8,000 g, and filtered through a 0.22 μm filter. *E. faecalis* was grown statically at 30°C in the presence of the extracted sterile intestinal juice added to THB liquid media at 1:1.

Larvae were weighed 7 d after inoculation with GFP–*E. faecalis* LX10. Pupation and adult emergence rates, whole cocoon weight, and cocoon shell weight were recorded after cocooning (*n* = 10).

### Growth Test at Different pH Levels and Measurement of Gut pH and Lactic Acid Production

To determine the growth rate of *E. faecalis* LX10 at different pH levels (6–11), the THB medium was dissolved in solutions with different pH levels in 100 mM NaHCO_3_/Na_2_CO_3_ buffer. *E. faecalis* LX10 cells were then immediately subjected to lactic acid measurement under different initial pH levels (*n* = 5, pH = 6–11) and different bacterial concentrations (*n* = 5, pH = 11, 10^3^, 10^5^, 10^7^, or 10^9^ CFU/mL) after 24 h of incubation using a Lactic Acid (LA) Content Assay Kit (D799099–0100, Sangon Biotech, Shanghai, China) (*n* = 5). A pH microelectrode (Unisense, Aarhus, Denmark) with a 20–30 μm tip diameter was used to measure the pH levels of fresh gut extracts obtained after silkworms had fed on a GFP–*E. faecalis* LX10 suspension (1 × 10^3^, 1 × 10^5^, 1 × 10^7^, or 1 × 10^9^ CFU/mL) for 5 d as previously described (Liu et al., 2016), with healthy germ-free *B. mori* larvae serving as a negative control (CK) (*n* = 10).

### Genome Sequencing, Assembly, and Annotation

The whole genome of *E. faecalis* LX10 was sequenced on the PacBio RSII platform at Majorbio Bio–pharm Technology Co., Ltd. (Shanghai, China). A total of 1,605 Mbp of high–quality reads were generated and filtered to remove sequences whose 5’ ends contained non–A, T, G, and C bases, sequences < 25 bp in length, sequences containing >10% Ns, and low-quality reads (≤ Q20). Short Oligonucleotide Analysis Package (SOAP) *de novo* V version 1.05 was used to generate contigs and scaffolds using different kmer sizes, and gaps were filled *via* the PCR amplification method (Liang et al., 2018; Xie et al., 2019). The raw dataset including both single and paired-end reads (average read length of 14,796 bp), is deposited at GenBank under BioProject and accession number ID: PRJNA810349 and CP092784-CP092785. Genes of strain LX10 were predicted by using Glimmer V3.02 software.^1^

The obtained genome component predictions included the prediction of protein-coding genes, tandem repetitive sequences, interspersed repeats, transposons, non-coding RNAs, prophages, and clustered regularly interspaced short palindromic repeat (CRISPR) sequences. The applied steps were as follows. The GeneMarkS program (Version 4.17) was used to identify related protein-coding genes (Besemer et al., 2001). Interspersed repetitive sequences were identified from genomic sequences using RepeatMasker,^2^ and tandem repeats were identified using Tandem Repeats Finder (TRF) (Benson, 1999; Saha et al., 2008). Virulence factors and antibiotic resistance genes were annotated based on the Virulence Factors Database (VFDB) (Liu et al., 2022) and the Comprehensive Antibiotic Resistance Database (CARD) (Alcock et al., 2020). PHAST was used for prophage prediction^3^ (Zhou et al., 2011). Transfer RNA (tRNA) and ribosomal RNA (rRNA) genes were detected with tRNAscan-SE V1.3.1^4^ and Barrnap V0.4.2,^5^ respectively (Lowe and Eddy, 1997). Small nuclear RNAs (snRNAs) were predicted by BLAST searches against the Rfam database (Nawrocki et al., 2015). The CRISPRFinder online tool^6^ was used for CRISPR identification (Grissa et al., 2007), and the genomic circle map was visualized by using Circos V0.64.^7^ Furthermore, the predicted gene functions were compared against the non-redundant protein NCBI non-redundant protein(NR), Clusters of Orthologous Groups (COG), Pfam, Swiss–Prot, Kyoto Encyclopedia of Genes and Genomes (KEGG), and Gene Ontology (GO) databases using BLASTp (BLAST 2.2.28 +) with an *E*-value cut off of 1e-5 (Ashburner et al., 2000).

### Overexpression and Purification of Bacteriocin and Measurement of Antimicrobial Activity

Total genomic DNA from *E. faecalis* LX10 was obtained and used as target DNA for PCR amplification of the bacteriocin gene (372 bp). The coding regions of bacteriocin (residues 22–126) were further cloned into the pET28a vector and transformed into *E. coli* BL21 (DE3) cells (TaKaRa, Dalian, China) by homologous recombination using the ClonExpress II One Step Cloning Kit (Vazyme, Nanjing, China). DE3 strains were cultured in LB medium, and expression was induced at an OD_600_ = 0.6 with 1 mM IPTG at 30°C for 6 h. DE3 cells were lysed in lysis buffer (10% glycerol, 25 mM Tris⋅HCl pH 8.0, 1% IGEPAL^®^ CA–630, 0.1 M sodium chloride, and 1 mM PMSF) by sonication for 15 min in an ice bath, followed by centrifugation at 10,000 × g for 30 min at 4°C. The hexahistidine-tagged bacteriocin protein was purified by using Ni–NTA agarose following the recommendations of the column manufacturer (QIAGEN, Germany). The bacteriocin protein was eluted in elution buffer (100 mM sodium chloride, 50 mM sodium phosphate, and 150 mM imidazole) in a small chromatography column (Sangon, Shanghai, China), and the elution fractions were collected. The collected fractions were then subjected to 15% (w/v) sodium dodecyl sulfate–polyacrylamide gel electrophoresis (SDS–PAGE).

The *E. faecalis* LX10 strain was cultured in THB (Hopebio, Qingdao, China) medium at 30°C for 36 h. The supernatant of *E. faecalis* LX10 was collected after centrifugation at 12,000 × g for 5 min and filtered through a 0.22 μm PVDF membrane (Millipore Corp., Billerica, MA) to obtain the cell-free fermentation liquid. After incubation at 4°C overnight, the crude proteins were precipitated with 60% saturated ammonium sulfate [(NH_4_)_2_SO_4_] and dialyzed through regenerated cellulose membrane tubing (3.5 kDa, Spectrum) in distilled water. Then, the crude proteins were concentrated *via* ultrafiltration with Amicon Ultra 3 kDa (Millipore Corp., Billerica, MA).

Antimicrobial activity of *E. faecalis* was evaluated using the agar well diffusion method (Zhang et al., 2017). For the determination of inhibitory spectra, bacteria strains, including the isolated *E. mundtii, E. casseliflavus*, *S. marcescens*, and *B. amyloliquefaciens*, *E.* coli, *Pseudomonas fulva*, *Staphylococcus sciuri*, and *Methylobacterium populi* type strains, were grown in the BHI media recommended by culture collections. Holes (10 mm) were cut from inoculated agar plates with sterile Pasteur pipettes and then filled with 100 μL of the tested culture filtrates. A 100 μL sample of the bacteriocin protein (2 mg/mL) or crude proteins (2 mg/mL) of *E. faecalis* was added to each well, and THB broth was used as a negative control. The inhibition zones were observed after 24–48 h of incubation at 30°C.

### Quantitative Real-Time PCR Validation of Genes

After feeding the silkworms GFP–*E. faecalis* LX10 (10^7^ CFU/mL), the expression levels of colonization-related genes [zinc ABC transporter substrate-binding protein (*znuA*), response regulator transcription factor (*BfmRS*), ABC transporter A (*patA*), ABC transporter B (*patB*), signal peptidase I (*lepB*), glycosyltransferase family 2 protein (*hssA*), bifunctional acetaldehyde-CoA/alcohol dehydrogenase (*adhE*), endocarditis and biofilm-associated pilus tip protein (*EbpA*), acetaldehyde dehydrogenase (*Lap*), cell surface protein precursor (*cspp*), CDP-glycerol glycerophosphotransferase family protein (*tagF*), ABC transporter ATP-binding protein (*ddpD*), serine/threonine protein kinase IreK (*prkC*), ATP-binding cassette domain-containing protein (*metN*) and cell surface protein (*csp*)] were determined at 0, 1, 3, 5, and 7 days using RT–qPCR (*n* = 5). Total RNA was isolated from the silkworm gut using a Promega Eastep^®^ Total RNA Super Extraction Kit (Beijing Biotec. Co., Ltd.) according to the supplier’s protocol. The RNA quality and concentration were measured with a NanoDrop spectrophotometer (Biodropsis BD–2000) and by gel electrophoresis (1% agarose gel). RNA of the desired quality (1 μg) was reverse transcribed into cDNA using HiScript^®^ II Q RT SuperMix R223–01 (Vazyme Biotech Co., Ltd.). RT–qPCR was performed according to a previously described of this study. All the results were normalized by using the 23 SrRNA housekeeping gene. The gene-specific primers used for transcript quantification are listed in the Supplementary Table 2. The 2^–^^ΔΔ*Ct*^ method was used to calculate the relative transcript abundance of the genes of interest.

### Statistical Analysis

Statistical analyses were carried out using GraphPad Prism (version 9.0). The appropriate test (parametric/non-parametric) was chosen after the data were checked for normality and homogeneity of variance. Analysis of variance (one–way ANOVA, Tukey’s *post-hoc* test) or Student’s *t*-test was performed to compare the results regarding gut bacterial quantification, *Enterococcus* quantification, and GFP–*E. faecalis* colonization, body mass, pupation and adult emergence rates, whole cocoon weight, cocoon shell weight, gut pH levels, lactic acid production, and gene expression changes across the experimental and control groups. The *P*-value for distinguishing the significance of the data was set at <0.05. All assays were repeated at least three times.

## Results

### Characteristics of *Enterococcus* Isolated From *Bombyx mori*

**FIGURE 1:**
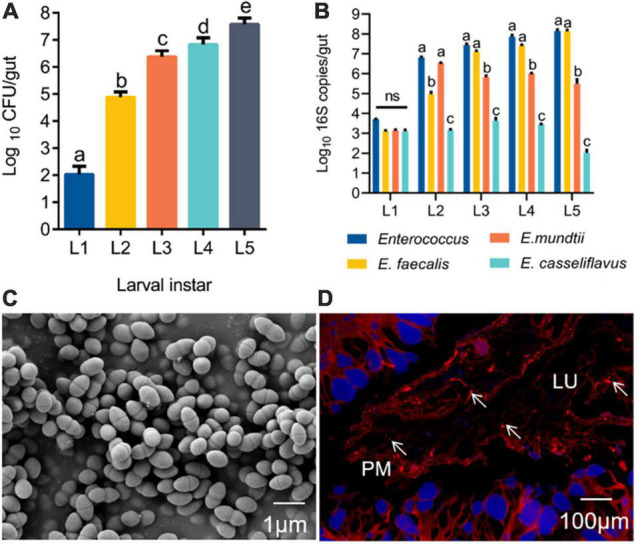
*Enterococcus* is widely found in the gut of silkworms. **(A)** Change in the *Enterococcus* population in the gut microbiota of *B. mori* larvae across different instars (L1, first instar; L2, second instar; L3, third instar; L4, fourth instar; L5, fifth instar). Proportional data are modeled using the mean number of CFUs counted (*n* = 10). **(B)** Absolute quantification (qPCR) of the total *Enterococcus*, *Enterococcus faecalis*, *Enterococcus mundtii*, and *Enterococcus casselifavus* 16S gene numbers in gut samples (L1-L5; mean with 95% confidence intervals). **(C)** Scanning electron microscopy (SEM) images of *E. faecalis*. Scale bars are 1 μm. **(D)** FISH with a Cy3–labeled *E. faecalis*-specific probe (red) shows a high density of bacterial cells adhered to the peritrophic membrane. lu, gut lumen; pm, peritrophic membrane; epithelium (blue, nuclei stained with DAPI). Statistically significant changes were determined by ANOVA and the *post hoc* Tukey honestly significant difference (HSD) test. Different letters represent statistically significant differences (*p* < 0.05, *n* = 10).

Before a silkworm metamorphoses into an adult, it goes through five larval instars (L1–L5), among which the 5th instar has the longest growth period (6–8 days). The gut population analysis revealed that *Enterococcu*s bacteria were present from the first instar to the fifth instar, and the number of *Enterococcus* progressively increased with *B. mori* age (Figure 1A). Based on phenotypic characteristics and 16S rRNA gene sequence analysis, three *Enterococcus* species were identified from the silkworm gut, including *E. faecalis* LX10, *E. mundtii* LX11, and *E. casseliflavus* LX12. *E. faecalis* LX10 was the most abundant of these strains on average relative to *E. mundtii* LX11 and *E. casseliflavus L*X12 isolated from silkworm samples of increasing age. The results for *E. faecalis* LX10 ranged from 1.30 × 10^3^ to 1.38 × 10^8^ 16S copies *per* larva, and this strain was consistently recovered from different batches of samples (Figure 1B). The obtained 16S rRNA gene sequences of *E. faecalis* LX10, *E. mundtii* LX11, and *E. casseliflavus* LX12 showed 100, 92, and 94% similarity, respectively, with available published sequences in GenBank. Additionally, the phylogenetic analysis revealed that these isolates from the silkworm gut clustered within the *Enterococcus* clade (Supplementary Figure 1). One representative strain, designated *E. faecalis* LX10 (as the dominant strain), was selected for further analysis. The cells of this bacterium are 0.5–1.0 μm in diameter and occur in pairs (Figure 1C). FISH with a Cy3-labeled *E. faecalis*-specific probe (red) showed large amounts of *E. faecalis* adhesioning to the PM of the *B. mori* gut epithelium (blue), where the bacterium formed a structural barrier (Figure 1D).

### GFP–*Enterococcus faecalis* Proliferates in the Gut of *Bombyx mori*

**FIGURE 2:**
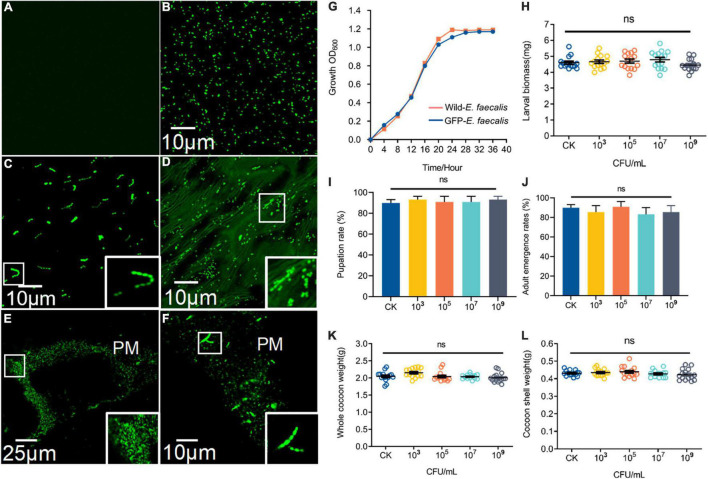
Characteristics and colonization of GFP–expressing *E. faecalis* in the *B. mori* intestinal tract. **(A)** Fluorescence micrographs of wild-type (CK) *E. faecalis* grown in THB. **(B)** Fluorescence micrographs of GFP–expressing *E. faecalis* under the control of pTRKH3–ermGFP. Scale bar = 10 μm. **(C)** GFP-*E. faecalis* grown for 12 h in the presence of intestinal juice and THB (1:1). Scale bar = 10 μm. **(D)** Fluorescent image of the gut contents of 5th-instar larvae after feeding on GFP–*E. faecalis* for 5 days. Scale bar = 10 μm. **(E)** Large clumps of GFP–*E. faecalis adhE*red to the peritrophic membrane of gut tissue. Scale bar = 25 μm. **(F)** *Enterococcus* with a chain–like structure was immobilized on the peritrophic membrane. Scale bar = 10 μm. **(G)** Shows the growth of these wild-type–*E. faecalis* and GFP–*E. faecalis.* The larval masses **(H)**, pupation **(I)**, whole cocoon weight **(J)** and adult emergence rates **(K)**, and cocoon shell weight **(L)** of normal and 10^3^, 10^6^, and 10^9^ CFU/mL *E. faecalis*–treated silkworms, respectively. Healthy silkworms treated with the same volume of sterilized water were used as the control group (CK). Different letters represent significant differences between groups according to Tukey’s honestly significant difference test (HSD, *p* < 0.05). Boxes show details.

The fluorescence intensity *E. faecalis* transformed with wild-type and pTRKH3–erm GFP *E. faecalis* was detected (Figures 2A,B). Interestingly, GFP–*E. faecalis* could switch from diplococcus form to occurring as chains in the presence of gut digestive juice (Figure 2C). In the intermediate and advanced stages (3–7 days) of ingestion, the fluorescent bacteria could be seen scattered among the gut contents of the 5th-instar larvae (Figure 2D). GFP bacteria accumulated around the PM, suggesting that there may be specific, stable cell attachment sites, and the bacteria seemed to aggregate in clusters and chains (Figures 2E,F). The growth curve analysis showed that wild-type *E. faecalis* and GFP–*E. faecalis* presented a typical “S”-shaped curve, indicating that the bacteria were suitable transgenic donors (Figure 2G). To examine whether *E. faecalis* was harmful to the host, we fed 5th-nstar larvae of *B. mori* GFP–tagged *E. faecalis*. The results showed no significant differences in larval biomass, pupation and adult emergence rates, whole cocoon weight, or cocoon shell weight between the control group and the GFP–*E*. *faecalis*–fed group (*p* > 0.05) (Figures 2H–L), suggesting that the bacteria were not toxic at the tested concentrations (10^3^ CFU/mL–10^9^ CFU/mL).

**FIGURE 3:**
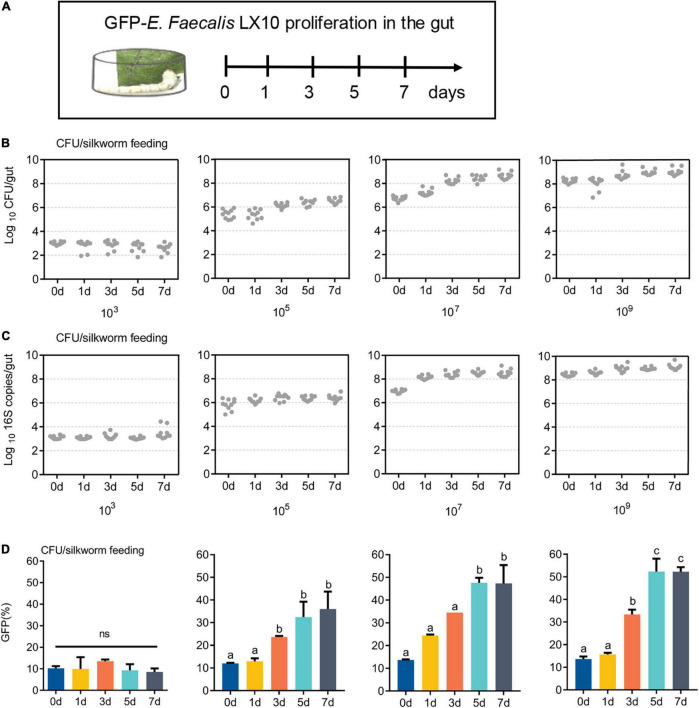
GFP–expressing *E. faecalis* stably persists and proliferates in the gut of *B. mori.* **(A)** First-instar (day 1) *B. mori* were inoculated with different concentrations of GFP–*E. faecalis* (10^3^, 10^6^, and 10^9^ CFU/mL). The silkworms were raised in cages, and feces were cleaned every 4 h, as shown in the scheme. **(B)** The number of CFUs in individual guts was assessed by plating at d 0, 1, 3, 5, and 7. Each dot represents one individual. **(C)** Absolute quantification of GFP–*E. faecalis* 16S gene numbers in gut samples (mean with 95% confidence intervals, *n* = 10). **(D)** A flow cytometry analysis (FACS) was performed on days 0, 1, 3, 5, and 7 to determine the percentage of GFP-positive cells. CFU, colony–forming unit. Statistically significant changes were determined by ANOVA and the *post hoc* Tukey honestly significant difference (HSD) test.

GFP-*E*. *faecalis* proliferation was determined by CFU, qPCR, and FACS analysis of silkworm guts kept in the same vial for 0, 1, 3, 5, or 7 days post feeding (Figure 3A). Over time, there was a statistically significant increase in bacterial levels in the silkworm gut, except at a low concentration (10^3^ CFU/silkworm feeding). The minimum and optimum feeding doses for colonization were found to be 10^5^ CFU/silkworm and 10^7^ CFU/silkworm, respectively (Figure 3B). On day 7, the total bacterial count (4.68 × 10^8^ CFU/gut) was significantly higher than that on day 0 (5.49 × 106 CFU/gut) (*t* = 17.58, *df* = 18, *p* < 0.0001), showing the long-term stability of GFP–*E. faecalis* LX10 after 10^7^ CFU/silkworm feeding. On the other hand, after 10^9^ CFU/silkworm feeding, although there was a statistically significant change between day 0 and day 7, the bacterial levels only increased from 1.82 × 10^8^ CFU/gut to 1.01 × 10^9^ CFU/gut (*t* = 6.843, *df* = 18, *p* < 0.0001) (Figure 3B). Independent experimental replicates based on qPCR using universal primers for the 16S rRNA gene showed similar results for all concentrations and time points (Figure 3C).

In addition, FACS analysis showed that the GFP–*E. faecalis* percentage was both dose and time dependent, and this phenomenon was more pronounced when the silkworms were treated fed with 10^7^CFU/silkworm (Figure 3D and Supplementary Figure 2). The percentage of GFP–*E. faecalis* increased from 13.65 to 46.5% during this period (0–7 days) (*t* = 197.6, df = 4, *p* < 0.0001). However, there was no difference in the number of GFP–*E. faecalis* between 0 and 7 days after 10^3^ CFU/silkworm feeding (10.16 vs. 8.5%) (*F* = 1.059, *p* = 0.4515, Figure 3D).

### General Features of the *Enterococcus faecalis* LX10 Genome

**FIGURE 4:**
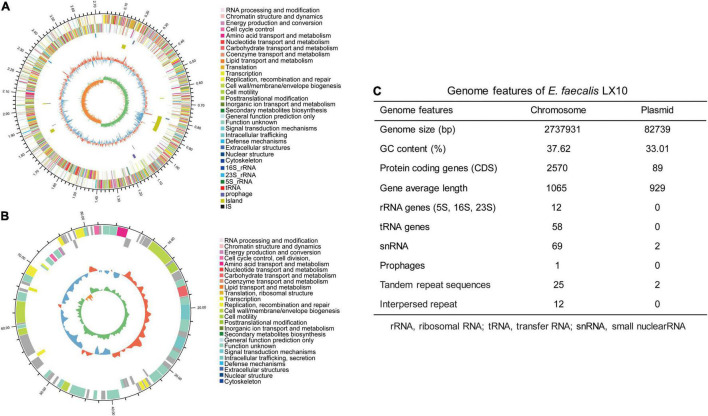
*E. faecalis* strain LX10 genome resources and characteristics. Circular representation of the chromosome **(A)** and plasmid **(B)** of *E. faecalis* strain LX10. The outer circle shows the size of the complete genome; second and third circles: sequences encoding the amino acids of proteins on the + and – strands, with different colors representing different COG functional classifications; fourth circle: rRNA and tRNA; fifth circle: GC content, where the outward red portion indicates that the GC content in this region is higher than the average GC content of the whole genome, and the inward blue portion indicates that the GC content in this region is lower than the whole-genome average, where the higher the peak value is, the greater the difference from the average GC content; and sixth circle: GC skew value (G–C/G + C). When the value is positive, the CDS is more likely to be transcribed from the positive chain; otherwise, the CDS is more likely to be transcribed from the negative chain. **(C)** Genome features of *E. faecalis* LX10.

In total, 1.61 billion high-quality bases were generated via the whole-genome shotgun sequencing approach. The *de novo* assembly of the sequences successfully generated a 2,737,931 bp circular chromosome with an average gene length of 1,065 bp (Figure 4A), and one plasmid was detected (Figure 4B). A total of 2,570 predicted coding sequences (CDSs), 58 tRNA genes, 12 rRNA operons, and 58 tRNA elements were identified in the genome (including the chromosome and plasmid) of *E. faecalis* LX10. One chromosomal region was identified as a prophage locus (Figure 4C).

The CDSs were compared with gene functions and metabolic pathways in the NR, GO, STRING, Swiss-Prot, COG, and KEGG databases. A total of 89 genes related to carbohydrate-active enzymes were annotated, including members of five CAZy families, comprising 46 glycoside hydrolases, 18 glycosyltransferases, 17 carbohydrate esterases, 7 auxiliary activities, and 1 polysaccharide lyase (Supplementary Figure 3A). A comprehensive genome-wide exploration of signal sequences, lipoprotein motifs, and other potential host cell components or binding motifs yielded an additional 217 putative surface-exposed proteins that may be associated with early colonization stages or virulence. We identified several genes (15) related to adhesin, defense, secretion, immune evasion, regulation, and iron acquisition proteins, some of which have been previously identified as colonization-related genes, whereas the other 202 genes showed no or vague functional predictions. And, the product of these genes are critical factors for host colonization (Supplementary Table 3 and Supplementary Figure 3B). Among the identified KEGG pathways, the genes involved in the global and overview maps (487 unigenes) and carbohydrate metabolism (224 unigenes) accounted for 39.22% of the predicted genes (Supplementary Figure 4A). GO analysis includes three ontologies: biological process (BP), cellular component (CC), and molecular function (MF). For example, under the CC and MF categories, the highest proportions of genes were annotated to the “integral component of membrane” (22.56%) and “ATP binding” (10.45%) terms (Supplementary Figure 4B). In the COG analysis, 2,317 COGs were classified into 19 functional categories. The analysis showed that most of the COG assigned ORFs fell under the category “Function unknown.” In the function known category, the highest and second-highest percentages of COGs were included in the carbohydrate transport and metabolism and transcription categories, which included 235 and 185 genes involved, respectively (Supplementary Figure 4C). In addition, a total of 558 genes (accounting for 20.98% of the total number of genes) were assigned to pathogenicity-related genes in the PHI database (Supplementary Figure 5A). The genes in the CARD database are genes whose presence confers resistance to antibiotics (Supplementary Figure 5B), such as lincosamide and streptogramin. The secretion system (Sec-SRP and type IV secretion system) of *E. faecalis* was checked based on the “Protein export (map03070)” pathway, and 10 key enzymes distributed in this pathway were identified (Supplementary Figure 6).

### Effect of pH on Cell Growth and Lactic Acid Production

**FIGURE 5:**
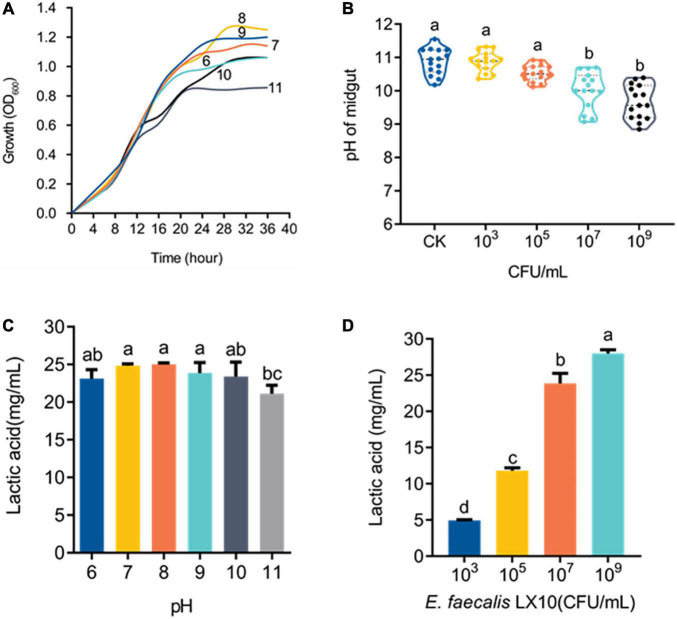
*E. faecalis* LX10 significantly reduces the intestinal pH and efficiently produces lactic acid. **(A)** Growth curves of the *E. faecalis* LX10 strain inoculated under pH-buffered conditions (pH: 6–11). **(B)** The pH of the germ-free silkworm gut after treatment with *E. faecalis* LX10 (CK, 10^3^, 10^6^, and 10^9^ CFU/mL). pH values are shown as the mean ± SE (*n* = 10). **(C)** Comparison of lactic acid production by *E. faecalis* LX10 (10^7^ CFU/mL) at different initial pH levels (*n* = 5, *p* > 0.05). **(D)** Lactic acid production by *E. faecalis* LX10 at different concentrations (10^3^, 10^5^, 10^7^, and 10^9^ CFU/mL; *n* = 5, *p* > 0.05). Different letters represent significant differences between groups according to Tukey’s honestly significant difference test (HSD, *p* < 0.05); ns not significant.

Cell growth was monitored under different pH-buffered (pH 6–11) conditions to monitor the effect of pH on *E. faecalis* LX10. The results showed that *E. faecalis* LX10 grew well, and the highest cell density was achieved within several hours, indicating that this strain is well adapted to the alkaline gut environment of silkworms (pH 9–11) (Figures 5A,B). In addition, *E. faecalis* inoculation resulted in a decrease in the gut pH [*F*_(4, 70)_ = 3.594, *p* < 0.0001], where the 10^9^ treatment resulted in the largest decrease relative to the CK group (9.00 vs. 10.83; *t* = 9.486, *df* = 28, *p* < 0.0001) (Figure 5B). Additionally, *E. faecalis* was able to produce lactic acid [*F*_(5, 24)_ = 3.037, *p* = 0.029] under most tested pH conditions (pH 6–11), with the highest titer being observed at the initial pH of 8 (25.21 g/L) (Figure 5C). There was a strong, positive correlation between lactic acid production and the *E. faecalis* concentration (10^3^–10^9^ CFU/mL, pH = 11) (Figure 5D). In addition, the functional annotation of the genome revealed several genomic features associated with lactic acid production in *E. faecalis* LX10 as well as genes related to the uptake of related sugars and initial metabolism, which included ABC transporter ATP–binding protein, ABC transporter permease, sugar ABC transporter permease, sugar ABC transporter ATP–binding protein, sugar ABC transporter, sugar-binding protein, alcohol dehydrogenase, acetate kinase, L–lactate dehydrogenase, acetate kinase, and acetaldehyde dehydrogenase genes (Supplementary Table 4).

### Antimicrobial Production and Bacterial Competition

**FIGURE 6:**
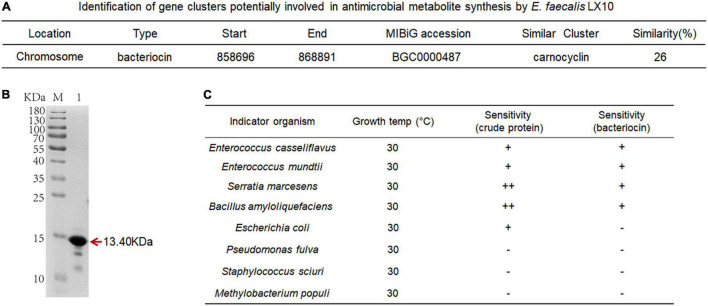
Characterization of bacteriocin and inhibitory activity. **(A)** Identification of gene clusters potentially involved in antimicrobial metabolite synthesis by *E. faecalis* LX10. **(B)** SDS–PAGE gel showing the reconstitution and purification of bacteriocin proteins. **(C)** Inhibition spectrum of the crude protein (2 mg/mL, 100 μL) from the cell-free culture supernatant and bacteriocin (2 mg/mL, 100 μL) produced by *E. faeca*lis LX10. *E. casseliflavus* LX10, *E. mundtii* LX11, *S. marcescens* LX12, *B. amyloliquefaciens*, *E. coli*, *P. fulva*, *S. sciuri*, and *M. populi as the* indicator. –, no inhibition; +, inhibition; ++ good inhibition.

The comparative analysis of genome sequences revealed a 14-gene cluster encoding a putative bacteriocin synthesis family that was enriched in *E. faecalis* isolates from the silkworm gut, suggesting that this bacteriocin may promote the colonization of indigenous microbiota (Figure 6A). The bacteriocin protein was purified by Ni–NATA affinity chromatography and examined by 15% SDS–PAGE. An evident band with a molecular weight of approximately 13.40 kDa was observed (lane 1 in Figure 6B and Supplementary Figure 7A), which was consistent with the predicted molecular mass of His–tagged bacteriocin.

The inhibition assays of *E. faecalis* were conducted by applying the plate confrontation test. *E. faecalis* LX10 showed a distinct antagonistic effect against the other five representative gut bacteria (*E. casseliflavus*, *E. mundtii*, *S. marcescens*, *B. amyloliquefaciens*, and *E. coli*) (Figure 6C). The crude proteins and bacteriocin protein of cell-free *E. faecalis* LX10 inhibited the growth of indicator bacteria and resulted in a clear inhibition zone (Supplementary Figure 7B).

### Genes Specifically Contributing to Gut Colonization

**FIGURE 7:**
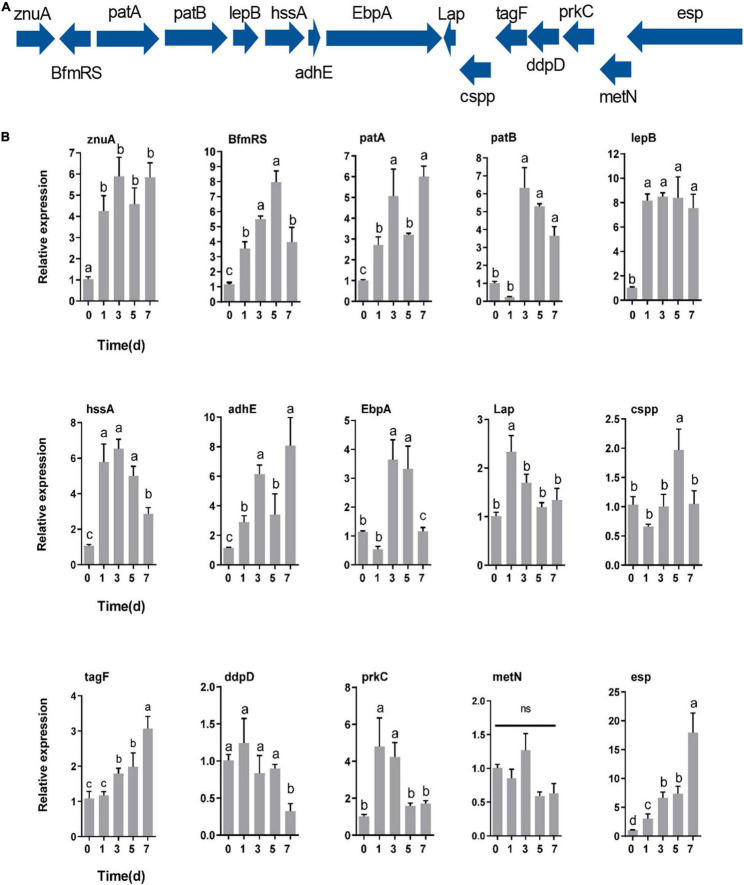
Genes specifically contributing to gut colonization. **(A)** Colonization-related gene expression at different times (0, 1, 3, 5, and 7 days) after inoculation with *E. faecalis* LX10 (10^7^ CFU/mL) by RT–qPCR. **(B)** The mean ± standard error of the mean (SEM) of experiments performed in quintuplicate, with 10 silkworms/experiment, are shown. Variation analysis was performed by one-way ANOVA followed by Tukey’s *post-hoc* test. Different letters represent significant differences at *p* < 0.05. Adhesin genes (*znuA*, *lepB*, *hssA*, *adhE*, *EbpA*, and *Lap*), defense genes (*cspp*, *esp*, and *tagF*), a regulatory gene (*BfmRS*), a secretion system gene (*prkC*), immune evasion genes (*patA* and *patB*), and iron acquisition genes (*ddpD* and *metN*).

RT–qPCR was utilized to determine how the r*esp*onse of *E. faecalis* gene expression after colonization of the host gut. The expression of genes encoding adhesin (*znuA*, *lepB*, *hssA*, *adhE*, *EbpA*, and *Lap*), defense (*cspp*, *tagF*, and *esp*), immune evasion (*patA* and *patB*), regulation (*BfmRS*), secretion gene (*prkC*) and iron acquisition (*ddpD* and *metN*) proteins was measured based on their colonization-related functions (Figure 7A). After oral feeding with *E. faecalis* LX10, six adhesin, three defense-related, two immune evasion-related, and two regulatory genes showed substantially increased expression (Figure 7B). In particular, compared with the level observed at 0 d, the expression of *BfmRS* (eightfold), *lepB* (eightfold) and *esp* (17-fold) reached extremely significant levels at 5 days (*t* = 10.64, *df* = 8, *p* = 0.0001), 3 days (*t* = 25.63, *df* = 8, *p* < 0.0001) and 7 days (*t* = 5.120, *df* = 8, *p* = 0.0009), respectively. Two iron acquisition protein genes showed decreased or no difference in gene expression over time (*p* > 0.05) (Figure 7B).

## Discussion

The successful colonization of the insect gut by symbiotic bacteria is an essential step in controlling beneficial microbe systems and the health status of the host (Paniagua Voirol et al., 2018; Sauers and Sadd, 2019). In this paper, we report that the level of *E. faecalis* is as high as 10^8^ copies in a normal 5th-instar larva gut, as confirmed by FISH analysis. In agreement with our results, previous studies have also found that *Enterococcus* is the most dominant bacterial genus in the gut microflora of silkworms (Xiang et al., 2007; Sun et al., 2016). Therefore, the dominant species, *E. faecalis*, is particularly likely to play a major role in facilitating the survival of its host in a changing environment during development.

Our results showed that the expression of GFP does not affect the physiology and fitness of *E. faecalis*, as strong fluorescence of recombinant GFP–*E. faecalis* was observed upon CLSM and FACS analyses. Similar results have been observed for *Lactobacillus reuteri* (Lizier et al., 2010) and *E. mundtii* (Teh et al., 2016). A recent study also showed that GFP expression did not negatively affect the bacterial function or survival of *E. coli* and *S. marcescens* (Wang S. et al., 2017; Chiapponi et al., 2020). In this study, GFP-tagged *E. faecalis* was introduced into the gut of *B. mori*, and it could stably persist and proliferate. Our recent study suggested that the minimum and optimum doses for colonization are 10^5^ CFU/mL to 10^7^ CFU/mL. Similarly, the minimum threshold for successful colonization for *Burkholderia* was shown to be 1.9 × 10^6^ symbionts in the stinkbug *Megacopta punctatissima* (Hosokawa et al., 2007; Kikuchi and Yumoto, 2013). In *Spodoptera littoralis*, the midgut CFU count was observed to range from 1.0 × 10^5^ during the fourth instar to 2.7 × 10^7^ in the fifth instar after feeding on fluorescent *E. mundtii* (Teh et al., 2016). This suggests that different host species may influence bacterial colonization. Haoyue × Jingsong strain was used in this study, which is the most widely used and popularized multifilament bivoltine *B. mori* strain in China. The stable colonization of the gut by *E. faecalis* requires introduction within a certain threshold range. This is probably because the food intake rates of silkworms affect the speed of microbe passage through the gut and, thus, the colonization efficiency *via* excretion (Smith et al., 2015; Zhang et al., 2022). In addition, we found that attachment to the gut appears to be required for *E. faecalis* to successfully colonize the PM. The PM of insects is composed of chitinous and glycoprotein structures that form a protective barrier against intestinal bacterial infection, abrasive food particles, digestive enzymes, and oral pathogens (Nakashima et al., 2018). Additionally, the PM compartmentalizes digestive processes, allowing efficient nutrient absorption and the reutilization of hydrolytic enzymes (Hegedus et al., 2009; Kuraishi et al., 2011; Rose et al., 2020). GFP bacteria accumulated around the PM, suggesting that there may be specific, stable cell attachment sites. Interestingly, GFP–*E. faecalis* may switch from diplococcus to chain form under treatment with intestinal juice. Previous work sowed that certain common intestinal molecules, bile acids, and lysozyme also increase chaining and biofilm formation in *E. faecalis*, thereby increasing the colonized surface area (Varahan et al., 2013; McKenney et al., 2019). In brief, the increased surface area of longer chains may advantageously increase adherence to the host, as bacteria must prevent themselves from being flushed off of system surfaces to achieve successful colonization (Weiser, 2013).

In addition, *E. faecalis* LX10 actively secretes the antimicrobial peptide bacteriocin, which may enhance its competitive fitness by inhibiting other competing gut bacteria. Bacteriocin production is a powerful means of shaping gut communities, and the interactions and transmission between a host and its gut microbiota determine colonization success (Stephens et al., 2015). Many factors, such as the gut environment (pH and osmotic pressure) (Kikuchi and Yumoto, 2013), host-species specificity (La Rivière et al., 2015), antimicrobial agents, and host or symbiont genotypes (Wang Q. et al., 2017), may determine colonization success. We speculate that the high competitiveness of *E. faecalis* in the gut may also be attributed to its ability to produce bacteriocins strengthening the competitive ability of *Enterococcus* against other bacteria (Cebrian et al., 2012). It has been reported that the pathogen *Helicobacter pylori* can be eradicated by autolysins secreted from *Lactobacillus acidophilus* and that *E. coli* O157:H7 can be inhibited by *Saccharomyces cerevisiae* in ruminal fluid (Lorca et al., 2001; Bach et al., 2003). In addition, the gut bacterium *E. mundtii* of *Spodoptera littoralis* can secrete stable class IIa bacteriocin, which facilitates the normal development of the gut microbiota over harmful gut invading bacteria (Shao et al., 2017). Bacteriocin exerts its activity against microbes by inhibiting the formation of cell walls or causing holes to form in the cell membrane, leading to the release of cytoplasm and the death of target bacteria (Scholl, 2017; Kranjec et al., 2020). Bacteriocin protects the bacterium against its own bacteriocin activity through the action of immune-related proteins, such as ABC transporter, LanI, and LanFEG proteins, which increase the colonization of probiotic bacteria vis cell–cell interactions in the digestive tract (McAuliffe et al., 2001).

*E. faecalis* LX10 can grow well under alkaline conditions and can decrease the pH of the gut by producing high levels of lactic acid. Many *Enterococcus* species are highly competitive due to their tolerance of a wide range of temperatures and pH levels (Abdel-Rahman et al., 2015; Kridsada et al., 2022). *E. faecalis* LX10 produces significantly higher levels of lactic acid (18.74–28.79 g/L) at different pH levels relative to species reported in other studies. For example, 9 *Enterococcus* spp. were selected from various sources, and their lactic acid production at 30°C and pH 10 ranged from 11.3 to 18.3 g/L (Yokaryo and Tokiwa, 2014). One reason that bacteria produce different amounts of acid may be their different isolation sources. Lactic acid may break down membrane-bound enzymes and lead to their metabolic assimilation through the lactic acid fermentation pathway to improve food digestibility (Kridsada et al., 2022). We speculate that lactic acid plays an important role in modulating the gut environment, which is conducive to maintaining the dynamic balance of the gut microbiota under alkaline conditions (Yuksekdag et al., 2021). Furthermore, genomic analysis revealed that the sugar uptake gene responsible for lactate production was dispersed across the chromosome and plasmid of *E. faecalis* LX10, reflecting its metabolic flexibility in efficient lactic acid production. For instance, the pentose phosphate pathway (PP pathway) and the phosphoketolase pathway (PK pathway) were previously reported to be the two main pathways for lactic acid fermentation (Okano et al., 2009; Shinkawa et al., 2011). Key enzymes involved in the PP and PK pathways, including ABC transporter permease and transketolase, were detected in *E. faecalis* LX10. Therefore, we speculated that the activity of related enzymes involved in lactic acid fermentation was quite stable under high-pH conditions, which might be one of the colonization mechanisms underlying specialized adaptation to the silkworm gut niche.

The successful colonization of *E. faecalis* LX10 led to drastically increased expression of most adhesin genes (*znuA*, *lepB*, *hssA*, *adhE*, *EbpA*, and *Lap*), defense-related genes (*cspp, tagF* and *esp*), regulation gene (*BfmRS*), secretion gene (*prkC*) and immune evasion-related genes (*patA* and *patB*), while the expression of iron acquisition protein-encoding genes (*ddpD* and *metN*) was largely unchanged or decreased relative to the expression levels in the gut on a genome-wide scale. *Enterococcus* species have been found to adhere to various extracellular matrix components or host cells using adhesins such as aggregation substances, hemagglutinin, MSCRAMM, and other virulence factors that contribute to the colonization of host tissues (Nallapareddy and Murray, 2008; Somarajan et al., 2015; Deng et al., 2019; Phillips et al., 2019). For example, *adhE* have been reported to play a role in adherence to host tissue cells for biofilm formation (Cobo Molinos et al., 2008; Crosby et al., 2020). *In vivo*, it has been shown that the *adhE* mutant shows a significantly attenuated biofilm formation ability in a rat endocarditis model and a murine urinary tract infection model (Nallapareddy et al., 2006; Singh et al., 2007). Another study showed that cell surface proteins (*cspp* and *esp*) contribute to cell colonization by significantly enhancing biofilm formation in the presence of and by attachment to tissue-based surfaces (Tendolkar et al., 2004). The PASTA kinase protein family contains a transmembrane Ser/Thr kinase (*prkC*) that is crucial for cell envelope integrity, adaptation to available nutrient sources, and resistance to antimicrobial agents that target the cell wall of *E. faecalis* (Hall et al., 2017). The deletion of *prkC* in *E. faecalis* results in a profound gastrointestinal tract colonization defect in antibiotic–naïve mice (Banla et al., 2018). Genes involved in core enterococcal and genome plasticity are key drivers of gut colonization.

## Conclusion

In conclusion, we identified a stable interaction between *B. mori* and *E. faecalis*, which will be useful for addressing important questions in the gut microbiota field that will lead to agricultural, economic, and environmental benefits. Moreover, the release of insects with stable symbionts in interventions may be effective against both pests (e.g., by improving the survival of sterile males) and pathogen vectors (e.g., by enhancing resistance to pathogens). Understanding what regulates gut colonization may be critical for the success of these approaches. Therefore, this new framework and concept for studying specific gut bacteria will contribute to the growing field of research on silkworm-microbe interactions.

## Data Availability Statement

The datasets presented in this study can be found in online repositories. The names of the repository/repositories and accession number(s) can be found below: https://www.ncbi.nlm.nih.gov/bioproject/PRJNA810349 and CP092784-CP092785.

## Author Contributions

XL and FZ designed the project and finalized the manuscript. XZ and HF performed the experiments. XZ, JH, and AM performed the data analysis. XZ drafted the manuscript with inputs from all authors.

## Conflict of Interest

The authors declare that the research was conducted in the absence of any commercial or financial relationships that could be construed as a potential conflict of interest.

## Publisher’s Note

All claims expressed in this article are solely those of the authors and do not necessarily represent those of their affiliated organizations, or those of the publisher, the editors and the reviewers. Any product that may be evaluated in this article, or claim that may be made by its manufacturer, is not guaranteed or endorsed by the publisher.

## Footnotes

1
http://www.cbcb.umd.edu/software/glimmer/

2
http://www.repeatmasker.org

3
http://phast.wishartlab.com

4
http://lowelab.ucsc.edu/tRNAscan-SE/index.html

5
http://vicbioinformatics.com/software.barrnap.shtml

6
http://crispr.i2bc.paris-saclay.fr/

7
http://circos.ca/

## References

[B1] Abdel-Rahman M. A. Tashiro Y. Zendo T. Sakai K. Sonomoto K. (2015). Enterococcus faecium QU 50: a novel thermophilic lactic acid bacterium for high-yield l-lactic acid production from xylose. *FEMS Microbiol. Lett.* 362 1–7. 10.1093/femsle/fnu030 25670701

[B2] Aksoy S. (2018). Insect gut microbiota: accessories to the bite. *Cell Host Microbe* 23 8–9. 10.1016/j.chom.2017.12.016 29324232

[B3] Alcock B. P. Raphenya A. R. Lau T. T. Y. Tsang K. K. Bouchard M. Edalatmand A. (2020). CARD 2020: antibiotic resistome surveillance with the comprehensive antibiotic resistance database. *Nucleic Acids Res.* 48 D517–D525. 10.1093/nar/gkz935 31665441 PMC7145624

[B4] Ashburner M. Ball C. A. Blake J. A. Botstein D. Butler H. Cherry J. M. (2000). Gene ontology: tool for the unification of biology. The gene ontology consortium. *Nat. Genet.* 25 25–29. 10.1038/75556 10802651 PMC3037419

[B5] Bach S. J. McAllister T. A. Veira D. M. Gannon V. P. J. Holley R. A. (2003). Effects of a Saccharomyces cerevisiae feed supplement on Escherichia coli O157:H7 in ruminal fluid in vitro. *Anim. Feed Sci. Technol.* 104, 179–189. 10.1016/s0377-8401(02)00325-5

[B6] Banla I. L. Kommineni S. Hayward M. Rodrigues M. Palmer K. L. Salzman N. H. (2018). Modulators of enterococcus faecalis cell envelope integrity and antimicrobial resistance influence stable colonization of the mammalian gastrointestinal tract. *Infect. Immun.* 86 e381–e317. 10.1128/IAI.00381-17 29038125 PMC5736811

[B7] Benson G. (1999). Tandem repeats finder: a program to analyze DNA sequences. *Nucleic Acids Res.* 27 573–580. 10.1093/nar/27.2.573 9862982 PMC148217

[B8] Besemer J. Lomsadze A. Borodovsky M. (2001). GeneMarkS: a self-training method for prediction of gene starts in microbial genomes. Implications for finding sequence motifs in regulatory regions. *Nucleic Acids Res.* 29 2607–2618. 10.1093/nar/29.12.2607 11410670 PMC55746

[B9] Bignell D. E. Roisin Y. Lo N. (2011). *Biology of Termites: A Modern Synthesis.* Cham: Springer.

[B10] Broderick N. A. Raffa K. F. Goodman R. M. Handelsman J. (2004). Census of the bacterial community of the gypsy moth larval midgut by using culturing and culture-independent methods. *Appl. Environ. Microbiol.* 70 293–300. 10.1128/AEM.70.1.293-300.2004 14711655 PMC321235

[B11] Cebrian R. Banos A. Valdivia E. Perez-Pulido R. Martinez-Bueno M. Maqueda M. (2012). Characterization of functional, safety, and probiotic properties of Enterococcus faecalis UGRA10, a new AS-48-producer strain. *Food Microbiol.* 30, 59–67. 10.1016/j.fm.2011.12.002 22265284

[B12] Chen B. Du K. Sun C. Vimalanathan A. Liang X. Li Y. (2018). Gut bacterial and fungal communities of the domesticated silkworm (Bombyx mori) and wild mulberry-feeding relatives. *ISME J.* 12 2252–2262. 10.1038/s41396-018-0174-1 29895989 PMC6092317

[B13] Chen B. Zhang N. Xie S. Zhang X. He J. Muhammad A. (2020). Gut bacteria of the silkworm *Bombyx mori* facilitate host resistance against the toxic effects of organophosphate insecticides. *Environ. Int.* 143:105886. 10.1016/j.envint.2020.105886 32623217

[B14] Chiapponi E. Henriot C. Bertrand X. Hocquet D. Bornette G. (2020). Using GFP-tagged *Escherichia coli* to investigate the persistence of fecal bacteria in vegetated wetlands: an experimental approach. *Antibiotics* 9:335. 10.3390/antibiotics9060335 32570743 PMC7344453

[B15] Ciche T. A. Kim K. S. Kaufmann-Daszczuk B. Nguyen K. C. Hall D. H. (2008). Cell invasion and matricide during *Photorhabdus luminescens* transmission by *Heterorhabditis bacteriophora* nematodes. *Appl. Environ. Microbiol.* 74 2275–2287. 10.1128/AEM.02646-07 18281425 PMC2293164

[B16] Cobo Molinos A. Abriouel H. Omar N. B. Lopez R. L. Galvez A. (2008). Detection of ebp (endocarditis- and biofilm-associated pilus) genes in enterococcal isolates from clinical and non-clinical origin. *Int. J. Food Microbiol.* 126 123–126. 10.1016/j.ijfoodmicro.2008.05.015 18571263

[B17] Crosby H. A. Tiwari N. Kwiecinski J. M. Xu Z. Dykstra A. Jenul C. (2020). The *Staphylococcus aureus* ArlRS two-component system regulates virulence factor expression through MgrA. *Mol. Microbiol.* 113 103–122. 10.1111/mmi.14404 31618469 PMC7175635

[B18] Deng L. Schilcher K. Burcham L. R. Kwiecinski J. M. Johnson P. M. Head S. R. (2019). Identification of key determinants of staphylococcus aureus vaginal colonization. *mBio* 10:e02321-19. 10.1128/mBio.02321-19 31874913 PMC6935855

[B19] Egert M. Wagner B. Lemke T. Brune A. Friedrich M. W. (2003). Microbial community structure in midgut and hindgut of the humus-feeding larva of *Pachnoda ephippiata* (Coleoptera: Scarabaeidae). *Appl. Environ. Microbiol.* 69 6659–6668. 10.1128/AEM.69.11.6659-6668.2003 14602626 PMC262301

[B20] Engel P. Moran N. A. (2013). The gut microbiota of insects - diversity in structure and function. *FEMS Microbiol. Rev.* 37 699–735. 10.1111/1574-6976.12025 23692388

[B21] Grissa I. Vergnaud G. Pourcel C. (2007). CRISPRFinder: a web tool to identify clustered regularly interspaced short palindromic repeats. *Nucleic Acids Res.* 35 W52–W57. 10.1093/nar/gkm360 17537822 PMC1933234

[B22] Hall C. L. Lytle B. L. Jensen D. Hoff J. S. Peterson F. C. Volkman B. F. (2017). Structure and dimerization of ireb, a negative regulator of cephalosporin resistance in *Enterococcus faecalis*. *J. Mol. Biol.* 429 2324–2336. 10.1016/j.jmb.2017.05.019 28551334 PMC5527686

[B23] Hammer T. J. Janzen D. H. Hallwachs W. Jaffe S. P. Fierer N. (2017). Caterpillars lack a resident gut microbiome. *Proc. Natl. Acad. Sci. U.S.A.* 114 9641–9646. 10.1073/pnas.1707186114 28830993 PMC5594680

[B24] Hegedus D. Erlandson M. Gillott C. Toprak U. (2009). New insights into peritrophic matrix synthesis, architecture, and function. *Annu. Rev. Entomol.* 54 285–302. 10.1146/annurev.ento.54.110807.090559 19067633

[B25] Hosokawa T. Kikuchi Y. Fukatsu T. (2007). How many symbionts are provided by mothers, acquired by offspring, and needed for successful vertical transmission in an obligate insect-bacterium mutualism? *Mol. Ecol.* 16 5316–5325. 10.1111/j.1365-294X.2007.03592.x 18028305

[B26] Kaiser W. Huguet E. Casas J. Commin C. Giron D. (2010). Plant green-island phenotype induced by leaf-miners is mediated by bacterial symbionts. *Proc. Biol. Sci.* 277 2311–2319. 10.1098/rspb.2010.0214 20356892 PMC2894905

[B27] Kikuchi Y. Fukatsu T. (2014). Live imaging of symbiosis: spatiotemporal infection dynamics of a GFP-labelled *Burkholderia symbiont* in the bean bug *Riptortus pedestris*. *Mol. Ecol.* 23 1445–1456. 10.1111/mec.12479 24103110 PMC4238818

[B28] Kikuchi Y. Hosokawa T. Fukatsu T. (2007). Insect-microbe mutualism without vertical transmission: a stinkbug acquires a beneficial gut symbiont from the environment every generation. *Appl. Environ. Microbiol.* 73 4308–4316. 10.1128/AEM.00067-07 17483286 PMC1932760

[B29] Kikuchi Y. Yumoto I. (2013). Efficient colonization of the bean bug *Riptortus pedestris* by an environmentally transmitted *Burkholderia symbiont*. *Appl. Environ. Microbiol.* 79 2088–2091. 10.1128/AEM.03299-12 23291548 PMC3592249

[B30] Koga R. Tsuchida T. Fukatsu T. (2009). Quenching autofluorescence of insect tissues for in situ detection of endosymbionts. *Appl. Entomol. Zool.* 44 281–291. 10.1303/aez.2009.281 33922110

[B31] Kranjec C. Ovchinnikov K. V. Gronseth T. Ebineshan K. Srikantam A. Diep D. B. (2020). A bacteriocin-based antimicrobial formulation to effectively disrupt the cell viability of methicillin-resistant *Staphylococcus aureus* (MRSA) biofilms. *NPJ Biofilms Microbiomes* 6:58. 10.1038/s41522-020-00166-4 33268776 PMC7710749

[B32] Kridsada U. Klongklaew A. Kodchasee P. Pamueangmun P. Shetty K. Khanongnuch C. (2022). Enterococci as dominant xylose utilizing lactic acid bacteria in Eri silkworm midgut and the potential use of *Enterococcus hirae* as probiotic for Eri culture. *Insect* 27:136. 10.3390/insects13020136 35206710 PMC8878294

[B33] Kuraishi T. Binggeli O. Opota O. Buchon N. Lemaitre B. (2011). Genetic evidence for a protective role of the peritrophic matrix against intestinal bacterial infection in *Drosophila melanogaster*. *Proc. Natl. Acad. Sci. U.S.A.* 108 15966–15971. 10.1073/pnas.1105994108 21896728 PMC3179054

[B34] La Rivière M. Garrabou J. Bally M. (2015). Evidence for host specificity among dominant bacterial symbionts in temperate gorgonian corals. *Coral Reefs* 34 1087–1098. 10.1007/s00338-015-1334-7

[B35] Liang X. Sun C. Chen B. Du K. Yu T. Luang-In V. (2018). Insect symbionts as valuable grist for the biotechnological mill: an alkaliphilic silkworm gut bacterium for efficient lactic acid production. *Appl. Microbiol. Biotechnol.* 102 4951–4962. 10.1007/s00253-018-8953-1 29627853

[B36] Liu B. Zheng D. Zhou S. Chen L. Yang J. (2022). VFDB 2022: a general classification scheme for bacterial virulence factors. *Nucleic Acids Res.* 50 D912–D917. 10.1093/nar/gkab1107 34850947 PMC8728188

[B37] Liu H. Chen B. Hu S. Liang X. Lu X. Shao Y. (2016). Quantitative proteomic analysis of germination of *Nosema bombycis* spores under extremely alkaline conditions. *Front. Microbiol.* 7:1459. 10.3389/fmicb.2016.01459 27708628 PMC5030232

[B38] Lizier M. Sarra P. G. Cauda R. Lucchini F. (2010). Comparison of expression vectors in Lactobacillus reuteri strains. *FEMS Microbiol. Lett.* 308 8–15. 10.1111/j.1574-6968.2010.01978.x 20455948 PMC7110086

[B39] Lorca G. L. Wadstrom T. Valdez G. F. Ljungh A. (2001). Lactobacillus acidophilus autolysins inhibit Helicobacter pylori in vitro. *Curr. Microbiol.* 42, 39–44. 10.1007/s002840010175 11116395

[B40] Lowe T. M. Eddy S. R. (1997). tRNAscan-SE: a program for improved detection of transfer RNA genes in genomic sequence. *Nucleic Acids Res.* 25, 955–967. 10.1093/nar/25.5.955 9023104 PMC146525

[B41] McAuliffe O. Ross R. P. Hill C. (2001). Lantibiotics: structure, biosynthesis and mode of action. *FEMS Microbiol. Rev.* 25 285–308. 10.1111/j.1574-6976.2001.tb00579.x 11348686

[B42] McKenney P. T. Yan J. Vaubourgeix J. Becattini S. Lampen N. Motzer A. (2019). Intestinal bile acids induce a morphotype switch in vancomycin-resistant enterococcus that facilitates intestinal colonization. *Cell Host Microbe* 25 695.e5–705.e5. 10.1016/j.chom.2019.03.008 31031170 PMC6939634

[B43] Nakashima K. Kimura S. Ogawa Y. Watanabe S. Soma S. Kaneko T. (2018). Chitin-based barrier immunity and its loss predated mucus-colonization by indigenous gut microbiota. *Nat. Commun.* 9:3402. 10.1038/s41467-018-05884-0 30143642 PMC6109156

[B44] Nallapareddy S. R. Murray B. E. (2008). Role played by serum, a biological cue, in the adherence of *Enterococcus faecalis* to extracellular matrix proteins, collagen, fibrinogen, and fibronectin. *J. Infect. Dis.* 197 1728–1736. 10.1086/588143 18462135 PMC2735109

[B45] Nallapareddy S. R. Singh K. V. Sillanpaa J. Garsin D. A. Hook M. Erlandsen S. L. (2006). Endocarditis and biofilm-associated pili of *Enterococcus faecalis*. *J. Clin. Invest.* 116 2799–2807. 10.1172/JCI29021 17016560 PMC1578622

[B46] Nawrocki E. P. Burge S. W. Bateman A. Daub J. Eberhardt R. Y. Eddy S. R. (2015). Rfam 12.0: updates to the RNA families database. *Nucleic Acids Res.* 43 D130–D137. 10.1093/nar/gku1063 25392425 PMC4383904

[B47] Okano K. Yoshida S. Tanaka T. Ogino C. Fukuda H. Kondo A. (2009). Homo-D-lactic acid fermentation from arabinose by redirection of the phosphoketolase pathway to the pentose phosphate pathway in L-lactate dehydrogenase gene-deficient Lactobacillus plantarum. *Appl. Environ. Microbiol.* 75 5175–5178. 10.1128/AEM.00573-09 19502433 PMC2725493

[B48] Paniagua Voirol L. R. Frago E. Kaltenpoth M. Hilker M. Fatouros N. E. (2018). Bacterial symbionts in lepidoptera: their diversity, transmission, and impact on the host. *Front. Microbiol.* 9:556. 10.3389/fmicb.2018.00556 29636736 PMC5881003

[B49] Phillips Z. N. Tram G. Seib K. L. Atack J. M. (2019). Phase-variable bacterial loci: how bacteria gamble to maximise fitness in changing environments. *Biochem. Soc. Trans.* 47 1131–1141. 10.1042/BST20180633 31341035

[B50] Rawls J. F. Mahowald M. A. Goodman A. L. Trent C. M. Gordon J. I. (2007). In vivo imaging and genetic analysis link bacterial motility and symbiosis in the zebrafish gut. *Proc. Natl. Acad. Sci. U.S.A.* 104 7622–7627. 10.1073/pnas.0702386104 17456593 PMC1855277

[B51] Rizzo M. A. Davidson M. W. Piston D. W. (2009). Fluorescent protein tracking and detection: fluorescent protein structure and color variants. *Cold Spring Harb. Protoc.* 2009:pdb.top63. 10.1101/pdb.top63 20150100

[B52] Robinson C. D. Klein H. S. Murphy K. D. Parthasarathy R. Guillemin K. Bohannan B. J. M. (2018). Experimental bacterial adaptation to the zebrafish gut reveals a primary role for immigration. *PLoS Biol.* 16:e2006893. 10.1371/journal.pbio.2006893 30532251 PMC6301714

[B53] Rose C. Casas-Sanchez A. Dyer N. A. Solorzano C. Beckett A. J. Middlehurst B. (2020). Trypanosoma brucei colonizes the tsetse gut via an immature peritrophic matrix in the proventriculus. *Nat. Microbiol.* 5 909–916. 10.1038/s41564-020-0707-z 32313202

[B54] Saha S. Bridges S. Magbanua Z. V. Peterson D. G. (2008). Empirical comparison of ab initio repeat finding programs. *Nucleic Acids Res.* 36 2284–2294. 10.1093/nar/gkn064 18287116 PMC2367713

[B55] Sauers L. A. Sadd B. M. (2019). An interaction between host and microbe genotypes determines colonization success of a key bumble bee gut microbiota member. *Evolution* 73 2333–2342. 10.1111/evo.13853 31584186 PMC7375351

[B56] Scholl D. (2017). Phage tail-like bacteriocins. *Annu. Rev. Virol.* 4 453–467. 10.1146/annurev-virology-101416-041632 28961412

[B57] Schretter C. E. (2020). Links between the gut microbiota, metabolism, and host behavior. *Gut Microbes* 11 245–248. 10.1080/19490976.2019.1643674 31345081 PMC7053934

[B58] Sellegounder D. Liu Y. Wibisono P. Chen C. H. Leap D. Sun J. (2019). Neuronal GPCR NPR-8 regulates *C. elegans* defense against pathogen infection. *Sci. Adv.* 5:eaaw4717. 10.1126/sciadv.aaw4717 31799388 PMC6867885

[B59] Shao Y. Chen B. Sun C. Ishida K. Hertweck C. Boland W. (2017). Symbiont-derived antimicrobials contribute to the control of the lepidopteran gut microbiota. *Cell Chem. Biol.* 24 66–75. 10.1016/j.chembiol.2016.11.015 28107652

[B60] Shinkawa S. Okano K. Yoshida S. Tanaka T. Ogino C. Fukuda H. (2011). Improved homo L-lactic acid fermentation from xylose by abolishment of the phosphoketolase pathway and enhancement of the pentose phosphate pathway in genetically modified xylose-assimilating *Lactococcus lactis*. *Appl. Microbiol. Biotechnol.* 91 1537–1544. 10.1007/s00253-011-3342-z 21637940

[B61] Siegel S. J. Weiser J. N. (2015). Mechanisms of Bacterial Colonization of the Respiratory Tract. *Annu. Rev. Microbiol.* 69 425–444. 10.1146/annurev-micro-091014-104209 26488280 PMC4760621

[B62] Singh K. V. Nallapareddy S. R. Murray B. E. (2007). Importance of the ebp (endocarditis- and biofilm-associated pilus) locus in the pathogenesis of *Enterococcus faecalis* ascending urinary tract infection. *J. Infect. Dis.* 195 1671–1677. 10.1086/517524 17471437 PMC2680192

[B63] Smith C. C. Snowberg L. K. Gregory Caporaso J. Knight R. Bolnick D. I. (2015). Dietary input of microbes and host genetic variation shape among-population differences in stickleback gut microbiota. *ISME J.* 9 2515–2526. 10.1038/ismej.2015.64 25909977 PMC4611514

[B64] Somarajan S. R. La Rosa S. L. Singh K. V. Roh J. H. Hook M. Murray B. E. (2015). The fibronectin-binding protein Fnm contributes to adherence to extracellular matrix components and virulence of *Enterococcus faecium*. *Infect. Immun.* 83 4653–4661. 10.1128/IAI.00885-15 26371130 PMC4645382

[B65] Stephens W. Z. Wiles T. J. Martinez E. S. Jemielita M. Burns A. R. Parthasarathy R. (2015). Identification of population bottlenecks and colonization factors during assembly of bacterial communities within the zebrafish intestine. *mBio* 6:e01163-15. 10.1128/mBio.01163-15 26507229 PMC4626852

[B66] Sun Z. Lu Y. Zhang H. Kumar D. Liu B. Gong Y. (2016). Effects of BmCPV infection on silkworm *Bombyx mori* intestinal bacteria. *PLoS One* 11:e0146313. 10.1371/journal.pone.0146313 26745627 PMC4706323

[B67] Swinfield T. J. Oultram J. D. Thompson D. E. Brehm J. K. Minton N. P. (1990). Physical characterisation of the replication region of the *Streptococcus faecalis* plasmid pAM beta 1. *Gene* 87 79–90. 2110101

[B68] Teh B. S. Apel J. Shao Y. Boland W. (2016). Colonization of the intestinal tract of the polyphagous pest *Spodoptera littoralis* with the GFP-tagged indigenous gut bacterium *Enterococcus mundtii*. *Front. Microbiol.* 7:928. 10.3389/fmicb.2016.00928 27379058 PMC4906056

[B69] Tendolkar P. M. Baghdayan A. S. Gilmore M. S. Shankar N. (2004). Enterococcal surface protein, Esp, enhances biofilm formation by *Enterococcus faecalis*. *Infect. Immun.* 72 6032–6039. 10.1128/IAI.72.10.6032-6039.2004 15385507 PMC517584

[B70] Varahan S. Iyer V. S. Moore W. T. Hancock L. E. (2013). Eep confers lysozyme resistance to *Enterococcus faecalis* via the activation of the extracytoplasmic function sigma factor SigV. *J. Bacteriol.* 195 3125–3134. 10.1128/JB.00291-13 23645601 PMC3697634

[B71] Viswanathan S. Williams M. E. Bloss E. B. Stasevich T. J. Speer C. M. Nern A. (2015). High-performance probes for light and electron microscopy. *Nat. Methods* 12 568–576. 10.1038/nmeth.3365 25915120 PMC4573404

[B72] Wang Q. Yang S. Liu J. Terecskei K. Abraham E. Gombar A. (2017). Host-secreted antimicrobial peptide enforces symbiotic selectivity in *Medicago truncatula*. *Proc. Natl. Acad. Sci. U.S.A.* 114 6854–6859. 10.1073/pnas.1700715114 28607058 PMC5495241

[B73] Wang S. Dos-Santos A. L. Huang W. Liu K. C. Oshaghi M. A. Wei G. (2017). Driving mosquito refractoriness to *Plasmodium falciparum* with engineered symbiotic bacteria. *Science* 357 1399–1402. 10.1126/science.aan5478 28963255 PMC9793889

[B74] Weiser J. N. (2013). The battle with the host over microbial size. *Curr. Opin. Microbiol.* 16 59–62. 10.1016/j.mib.2013.01.001 23395472 PMC3622179

[B75] Wellinghausen N. Bartel M. Essig A. Poppert S. (2007). Rapid identification of clinically relevant Enterococcus species by fluorescence in situ hybridization. *J. Clin. Microbiol.* 45, 3424–3426. 10.1128/JCM.00861-07 17670922 PMC2045329

[B76] Xiang H. Li M. Zhao Y. Zhao L. Zhang Y. Huang Y. (2007). Bacterial community in midguts of the silkworm larvae estimated by PCR/DGGE and 16S rDNA gene library analysis. *Jiangxi Science* 50 222–233.

[B77] Xiao X. Yang L. Pang X. Zhang R. Zhu Y. Wang P. (2017). A Mesh-Duox pathway regulates homeostasis in the insect gut. *Nat. Microbiol.* 2:17020. 10.1038/nmicrobiol.2017.20 28248301 PMC5332881

[B78] Xie S. Vallet M. Sun C. Kunert M. David A. Zhang X. (2019). Biocontrol potential of a novel endophytic bacterium from mulberry (morus) tree. *Front. Bioeng. Biotechnol.* 7:488. 10.3389/fbioe.2019.00488 32039187 PMC6990687

[B79] Yeruva T. Vankadara S. Ramasamy S. Lingaiah K. (2020). Identification of potential probiotics in the midgut of mulberry silkworm, *Bombyx mori* through metagenomic approach. *Probiotics Antimicrob. Proteins* 12 635–640. 10.1007/s12602-019-09580-3 31401774

[B80] Yokaryo H. Tokiwa Y. (2014). Isolation of alkaliphilic bacteria for production of high optically pure L-(+)-lactic acid. *J. Gen. Appl. Microbiol.* 60 270–275. 10.2323/jgam.60.270 25742979

[B81] Yuksekdag Z. Ahlatci N. S. Hajikhani R. Darilmaz D. O. Beyatli Y. (2021). Safety and metabolic characteristics of 17 *Enterococcus faecium* isolates. *Arch. Microbiol.* 203 5683–5694. 10.1007/s00203-021-02536-8 34468805

[B82] Zhang F. Sun X. X. Zhang X. C. Zhang S. Lu J. Xia Y. M. (2017). The interactions between gut microbiota and entomopathogenic fungi: a potential approach for biological control of *Blattella germanica* (L.). *Pest Manag. Sci.* 74 438–447. 10.1002/ps.4726 28888066

[B83] Zhang X. Feng H. He J. Liang X. Zhang N. Shao Y. (2022). The gut commensal bacterium *Enterococcus faecalis* LX10 contributes to defending against *Nosema bombycis* infection in *Bombyx mori*. *Pest Manag. Sci.* 78 2215–2227. 10.1002/ps.6846 35192238 PMC9314687

[B84] Zhou Y. Liang Y. Lynch K. H. Dennis J. J. Wishart D. S. (2011). PHAST: a fast phage search tool. *Nucleic Acids Res.* 39 W347–W352. 10.1093/nar/gkr485 21672955 PMC3125810

