# Supplementary material for: Features and Colonization Strategies of Enterococcus faecalis in the Gut of Bombyx mori
Source: Front Microbiol. 2022 Jun 24;13:921330. doi: 10.3389/fmicb.2022.921330 (PMC9263704; doi:10.3389/fmicb.2022.921330)
Supplement: Supplementary file 1 [file Data_Sheet_1.docx]

**Supplementary Table S1** The bacterial strains and plasmids used in this study

| **Strain or plasmid** | **Relevant properties** | **Reference or source** |
| --- | --- | --- |
| Strain |  |  |
| *E. faecalis* LX10 | Isolated from the intestine of *B. mori* | Laboratory collection |
| *E. coli* DH5α | Transformed bacteria in stab cultures | Takara |
| *E. casseliflavus* | Isolated from the intestine of *B. mori* | Laboratory collection |
| *E. mundtii* | Isolated from the intestine of *B. mori* | Laboratory collection |
| *S. marcescens* | Isolated from the intestine of *B. mori* | Laboratory collection |
| *B. amyloliquefaciens* | Isolated from the intestine of *B. mori* | Laboratory collection |
| *Methylobacterium populi* | Isolated from the intestine of *B. mori* | Laboratory collection |
| *Pseudomonas fulva* | Isolated from the intestine of *B. mori* | Laboratory collection |
| *Staphylococcus sciuri* | Isolated from the intestine of *B. mori* | Laboratory collection |
| *Escherichia coli* | Isolated from the intestine of *B. mori* | Laboratory collection |
| Plasmid |  |  |
| pTRKH3 | *E. coli*–lactic acid bacteria (LAB) shuttle vector | O’Sullivan and Klaenhammer, 1993 |
| pTRKH3–ermGFP | pTRKH3 derivative containing gfp gene downstream of ermB promoter | Addgene plasmid 27169 |

**Supplementary** **Table S2** Primers used in this study.

| **Target gene** | **Primer** | **Sequence（F/R）** |
| --- | --- | --- |
| 16S rRNA | 27F  1492R | AGAGTTTGATCCTGGCTCAG  GGTTACCTTGTTACGACTT |
| DB200 | F  R | CGGYCCAGACTCCTACGGG  TTACCGCGGCTGCTGGCAC |
| *E. faecalis* | F  R | CCCGAGTGCTTGCACTCAATTGG  CCGTCAAGGGATGAACATTTTAC |
| *E. mundtii* | F  R | AGCTTGCTCCACCGGAAAAAGA  ATCCATCAGCGACACCSKAA |
| *E. casselifavus* | F  R | CACTATTTTCCGCATGGAAGAAAG  CCGTCAAGGGATGAACATTTTAC |
| GFP | F  R | TCGGAATTCATGAGTAAAGGAGAAGAA  TCAGGATCCTTATTTGTATAGTTCATCC |
| znuA | F  R | TTTTATCCGATGTACGACT  AATAACATTCGGAACCCAA |
| BfmRS | F  R | ACCGCTATCAATTGTTAAGCA  AAACCTCAGTATCTAAGGCAT |
| patA | F  R | AAATGTCATTATGATTGCGTTG  TTTGACGACACGAATGCCTA |
| patB | F  R | TAATCTTTTCAGTACGGGACA  GATCCGCATTTAGAATCGTT |
| lepB | F  R | AGTCTTAGTACAACGGACGAA  CATTATCGCTTGCTATAGGTTC |
| hssA | F  R | TTATGATGCGGACAATACACC  TAATTGAAAACGACCGGCTTG |
| adhE | F  R | ACAAATGCGATTTTATTACCAC  GTTCATGTCAATTCCGACT |
| EbpA | F  R | TTACGGAACACAATTTACGAA  CATGTATTTCAATCCCACGTT |
| Lap | F  R | AGGCACAGCAAGTATTAGCAA  ACTACTTTTGAAGCGAAAGCA |
| cspp | F  R | TATCAAATTGCTCCTAACTCC  TTGATTAATTGCTTTCGCTTG |
| tagF | F  R | TTTCACGCTATATTTATCCTG  GAATTATTGTCAACACGTTT |
| ddpD | F  R | CCATTCAGGCACAAATTCTCG  CCATTTCCGCAACTACACC |
| prkC | F  R | CTGGTTCACCCGAATATCGTC  AAATTTGTTGCGTAATGTCCAC |
| metN | F  R | CGACTATTACATAAGACGCATC  ATTCGCCAATTAACTGACC |
| esp | F  R | CACCCGCTGATGTTACCAC  AGTTTCCTTGATCATCCGTTG |
| 23SrRNA | F  R | CCTATCGGCCTCGGCTTAG  AGCGAAAGACAGGTGAGAATCC |

**Supplementary** **Table S3** The features and functions of colonization-related genes

| **Locus** | **Description** | **Gene Name** | **Location** | **Function** |
| --- | --- | --- | --- | --- |
| gene0048 | zinc ABC transporter substrate-binding protein | znuA | Chromosome: 53790-54743 | Adherence |
| gene0267 | response regulator transcription factor | BfmRS | Chromosome: complement(255375-256067) | Regulation |
| gene0514 | ABC transporter | patA | Chromosome: 525699-527414 | Immune evasion |
| gene0515 | ABC transporter | patB | Chromosome: 527414-529183 | Immune evasion |
| gene0575 | signal peptidase I | lepB | Chromosome: 589554-590129 | Adherence |
| gene0605 | glycosyltransferase family 2 protein | hssA | Chromosome: 630248-631510 | Adherence |
| gene0616 | bifunctional acetaldehyde-CoA/alcohol dehydrogenase | adhE | Chromosome: 641930-644527 | Adherence |
| gene0918 | endocarditis and biofilm-associated pilus tip protein | EbpA | Chromosome: 942738-946049 | Adherence |
| gene1381 | acetaldehyde dehydrogenase (acetylating) | Lap | Chromosome: complement(1439715-1441193) | Adherence |
| gene1560 | cell surface protein precursor | cspp | Chromosome: complement(1620425-1621474) | Defensive |
| gene1743 | CDP-glycerol glycerophosphotransferase family protein | tagF | Chromosome: complement(1828400-1829551) | Defensive |
| gene2367 | ABC transporter ATP-binding protein | ddpD | Chromosome: 1985453-1986079 | Iron uptake system |
| gene2377 | serine/threonine protein kinase IreK | prkC | Chromosome: complement(2521280-2519124) | Secretion system |
| gene2455 | ATP-binding cassette domain-containing protein | metN | Chromosome: complement(2205513-2206139) | Iron uptake system |
| gene2555 | cell surface protein | esp | Chromosome: complement(2715704-2720620) | Defensive |
