# Supplementary material for: Features and Colonization Strategies of Enterococcus faecalis in the Gut of Bombyx mori
Source: Front Microbiol. 2022 Jun 24;13:921330. doi: 10.3389/fmicb.2022.921330 (PMC9263704; doi:10.3389/fmicb.2022.921330)
Supplement: Supplementary file 2 [file Data_Sheet_2.pdf]

Supplementary Material

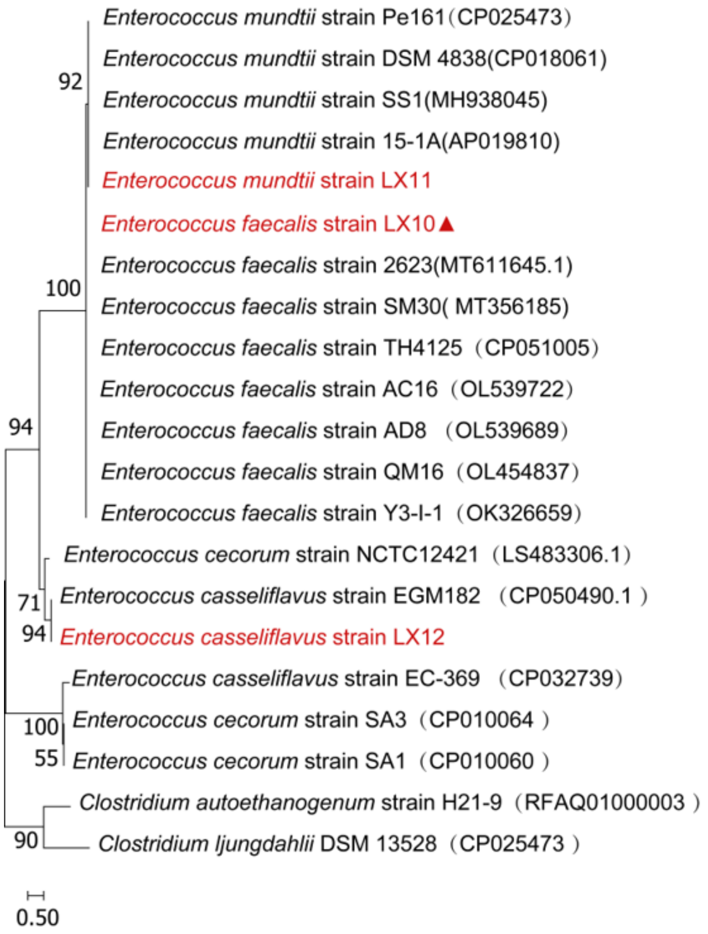

Supplementary Figure 1. Phylogenetic tree indicating the relationships of *Enterococcus* strain (*E. faecalis*, *E. mundtii*, and *E. casselifavus*) 16S rRNA gene sequences with sequences of related bacterial species from GenBank. Phylogenetic trees were reconstructed with MEGA 11 by applying the neighbor-joining method. The applied parameters were as follows: bootstrap method (1,000 replicates), substitutions to include (crossovers and transitions), uniform rates, Kimura 2-parameter model and pairwise deletion.

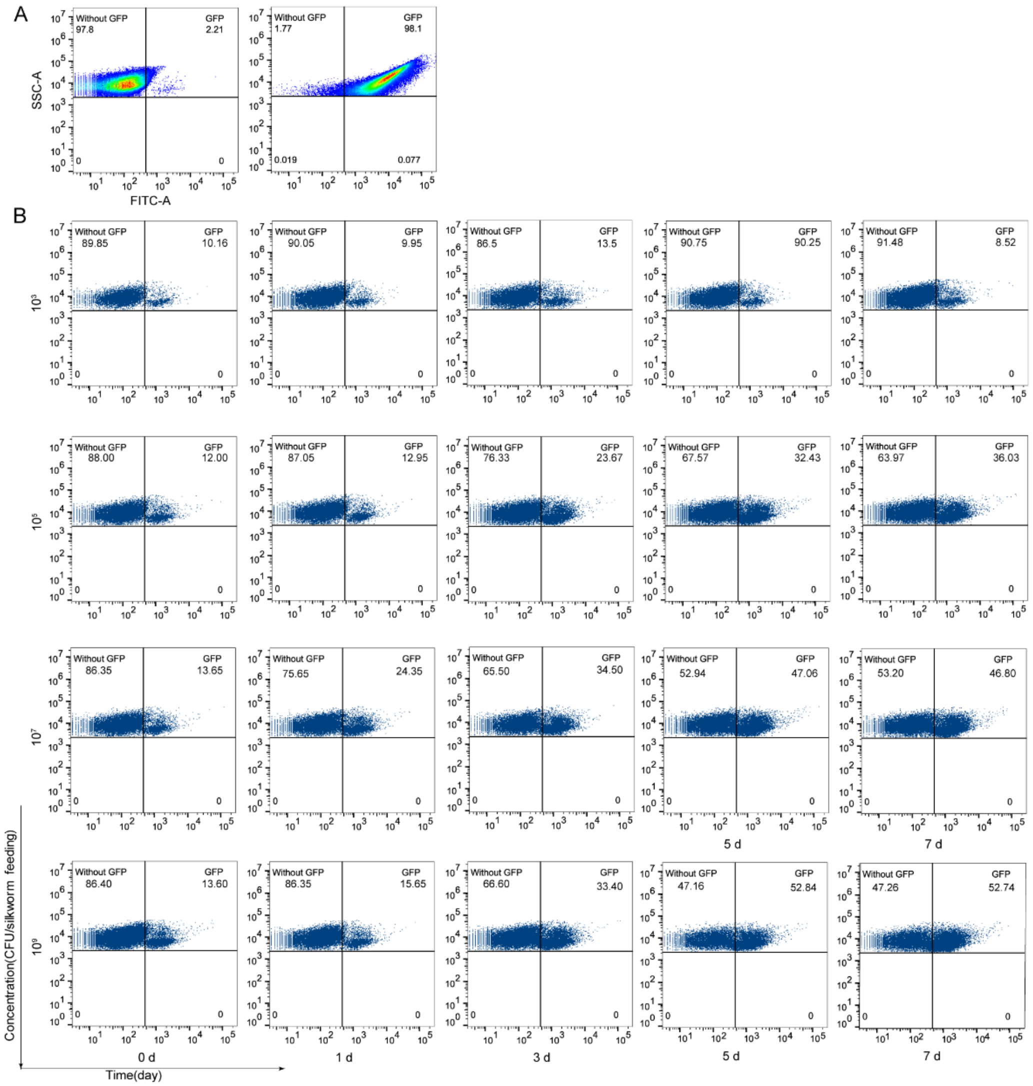

Supplementary Figure 2. The percentage of GFP-positive cells was quantified by flow cytometry on days 0, 1, 3, 5, and 7. Sorting profiles of *E. faecalis*–pTRKH3 from the negative control (*E. faecalis* without GFP) and positive control (*E. faecalis* with GFP) **(A)** of THB broth-grown fluorescent *E. faecalis*–pTRKH3 or the gut homogenate after feeding on GFP–*E. faecalis* (10^3, 10^6, and 10^9 CFU/mL) (**B**) X–axis: GFP fluorescence. Y–axis: Pacific blue fluorescence (empty channel used to facilitate gating). The number within the gate represents the percentage of GFP–positive or *E. faecalis* without GFP events among the total events.

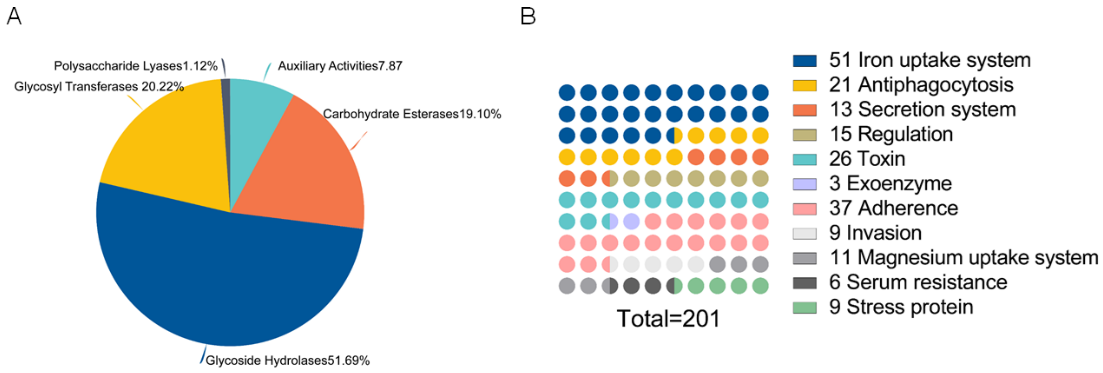

Supplementary Figure 3. (**A**) Gene count distributions of carbohydrate-active enzyme (CAZy) families of *E. faecalis* LX10 isolated from the silkworm gut: glycoside hydrolases, glycosyl transferases, carbohydrate binding modules, auxiliary activities and polysaccharide lyases. (**B**) *E. faecalis* LX10 virulence-related genes predicted in the LX10 genome.

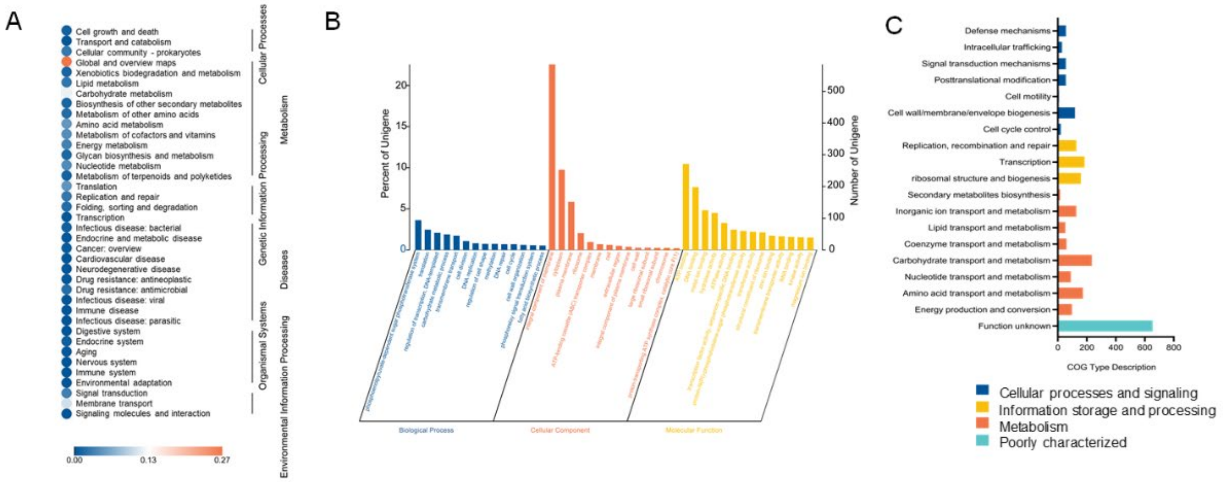

Supplementary Figure 4. (**A**) Kyoto Encyclopedia of Genes and Genomes (KEGG) pathway enrichment analysis; (**B**) Gene Ontology (GO) functional annotation; (**C**) Clusters of Orthologous Groups of proteins (COG) functional classification of *E. faecalis* LX10 proteins.

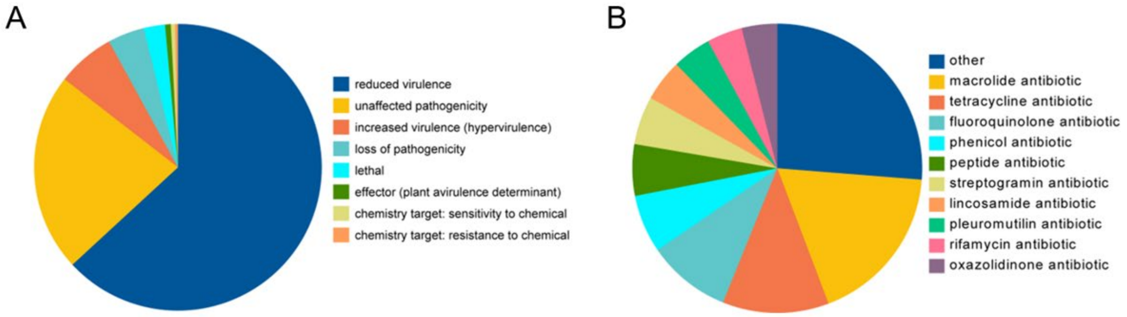

Supplementary Figure 5. (**A**) Pathogen host interaction (PHI) annotations and (**B**) resistance genes.

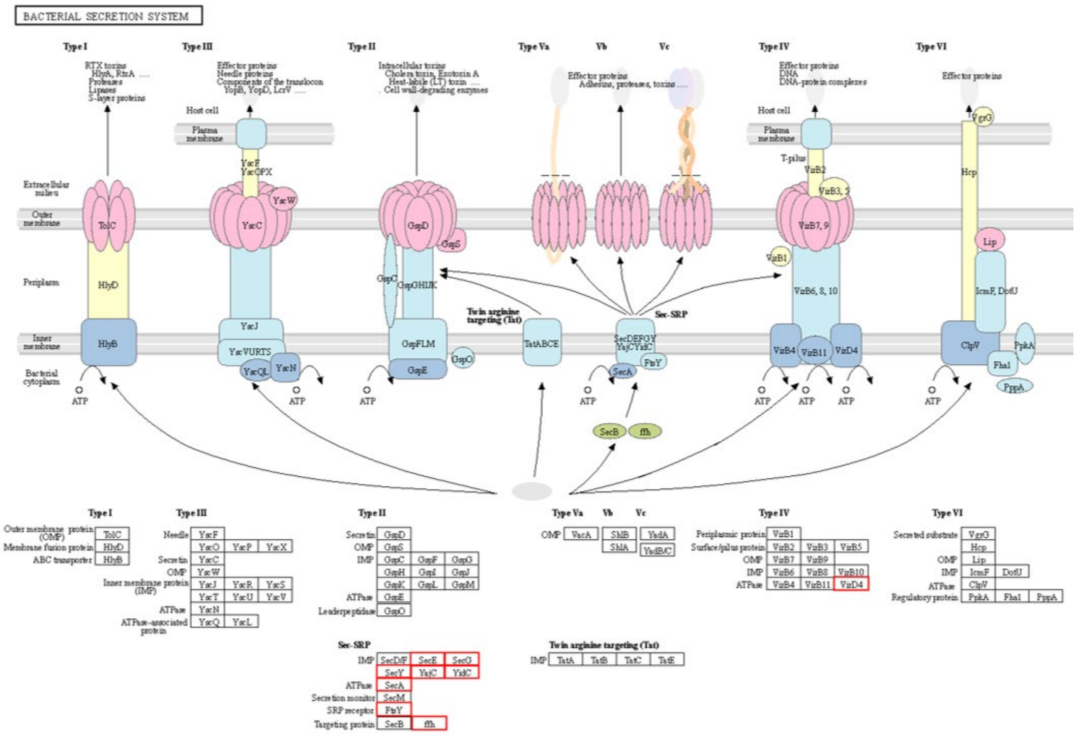

Supplementary Figure 6. "Protein export" pathway of *E. faecalis* LX10. A red box indicates existing homologous genes of the enzyme, while a white box indicates nonhomologous genes. The photo was created by KEGG Mapper.

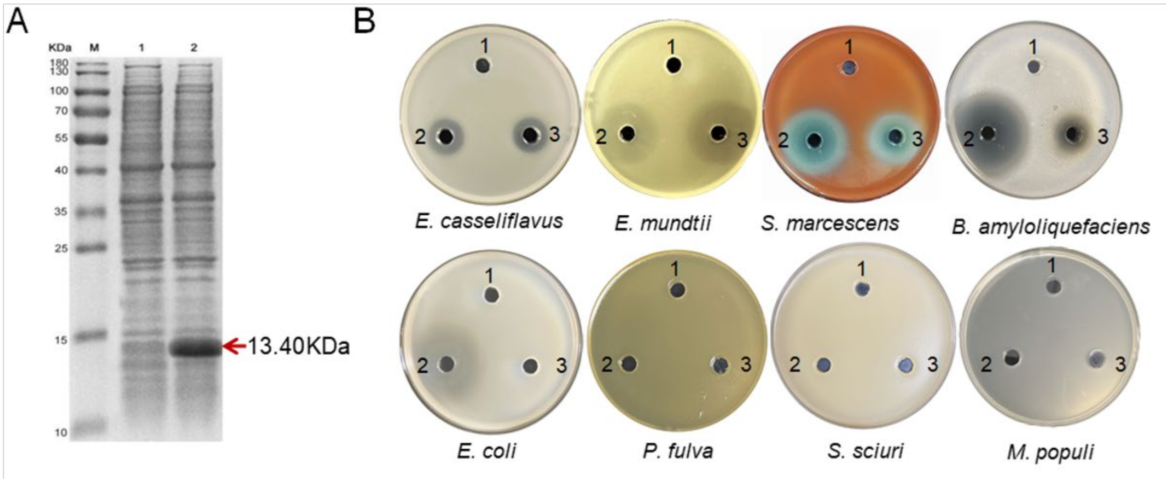

Supplementary Figure 7. Characterization of bacteriocin and inhibitory activity. (**A**) The recombinant bacteriocin protein was separated by SDS–PAGE from pET-28a (+) with *E. coli* BL21. Lane M: standard protein molecular weight marker (10-180 kDa); Lane 1: negative control (without induction); lane 2: bacteriocin bands (red arrow). (**B**) Typical results of agar diffusion assays using *E. casseliflavus* LX10 *and E. mundtii* LX10, *S. marcescens*, *B. amyloliquefaciens*, *E. coli*, *P. fulva*, *S. sciuri,* and *M. populi* as indicators. 1, THB medium was used as a negative control (100 µL). 2, Crude protein (2 mg/mL, 100 µL) from the cell-free culture supernatant of *E. faecalis* LX10; 3, purified bacteriocin (2 mg/mL, 100 µL) from *E. coli*. Representative results of at least five independent experiments are shown.
